# Unlocking the agro-physiological potential of wheat rhizoplane fungi under low P conditions using a niche-conserved consortium approach

**DOI:** 10.1093/jxb/eraf042

**Published:** 2025-02-26

**Authors:** Brahim Benbrik, Tessa E Reid, Dounia Nkir, Hicham Chaouki, Yassine Aallam, Ian M Clark, Tim H Mauchline, Jim Harris, Mark Pawlett, Abdellatif Barakat, Zineb Rchiad, Adnane Bargaz

**Affiliations:** AgroBiosciences Program, College of Agriculture and Environmental Sciences, Mohammed VI Polytechnic University, Ben Guerir, Morocco; Sustainable Soils and Crops, Rothamsted Research, Harpenden, UK; AgroBiosciences Program, College of Agriculture and Environmental Sciences, Mohammed VI Polytechnic University, Ben Guerir, Morocco; AgroBiosciences Program, College of Agriculture and Environmental Sciences, Mohammed VI Polytechnic University, Ben Guerir, Morocco; AgroBiosciences Program, College of Agriculture and Environmental Sciences, Mohammed VI Polytechnic University, Ben Guerir, Morocco; Sustainable Soils and Crops, Rothamsted Research, Harpenden, UK; Sustainable Soils and Crops, Rothamsted Research, Harpenden, UK; Environment and Agrifood, Faculty of Engineering and Applied Sciences, Cranfield University, Cranfield MK43 0AL, UK; Environment and Agrifood, Faculty of Engineering and Applied Sciences, Cranfield University, Cranfield MK43 0AL, UK; AgroBiosciences Program, College of Agriculture and Environmental Sciences, Mohammed VI Polytechnic University, Ben Guerir, Morocco; IATE, Université de Montpellier, INRAE, Agro Institut. 2, Place Pierre Viala, 34060 Montpellier, France; Biosciences Division, CoreLabs, Mohammed 6 Polytechnic University, Ben Guerir, Morocco; AgroBiosciences Program, College of Agriculture and Environmental Sciences, Mohammed VI Polytechnic University, Ben Guerir, Morocco; University of Ghent, Belgium

**Keywords:** Fungal consortia, fungal diversity, niche-conserved, phosphate, plant growth promotion, rhizoplane

## Abstract

Plant growth-promoting fungi (PGPF) hold promise for enhancing crop yield. This study delves into the fungal diversity of the wheat rhizoplane across seven Moroccan agricultural regions, employing a niche-conserved strategy to construct fungal consortia (FC) exhibiting higher phosphorus (P) acquisition and plant growth promotion. This study combined culture-independent and culture-dependent methods exploring taxonomic and functional diversity in the rhizoplane of wheat plants obtained from 28 zones. Twenty fungal species from eight genera were isolated and confirmed through internal transcribed spacer (ITS) Sanger sequencing. P solubilization (PS) capacity was assessed for individual species, with *Talaromyces* sp. (F_11_) and *Rhizopus arrhizus* CMRC 585 (F_12_) exhibiting notable PS rates, potentially due to production of organic acids such as gluconic acid. PGPF traits and antagonism activities were considered when constructing 28 niche-conserved FC (using isolates from the same zone), seven intra-region FC (different zones within a region), and one inter-region FC. Under low P conditions, *in planta* inoculation with niche-conserved FC (notably FC_14_ and FC_17_) enhanced growth, physiological parameters, and P uptake of wheat, in both vegetative and reproductive stages. FC_14_ and FC_17_, composed of potent fungi such as F_11_ and F_12_, demonstrated superior plant growth benefits compared with intra- and inter-region constructed FC. Our study underscores the efficacy of the niche-conserved strategy in designing synthetic fungal community from isolates within the same niche, proving significant agro-physiological potential to enhance P uptake and plant growth of wheat.

## Teaser

The present study demonstrated the agro-physiological potential of rhizoplane niche-conserved fungal consortia to enhance wheat plant growth and nutrient acquisition under low P conditions, thus contributing to sustainable crop production.

## Introduction

Wheat is one of the most important cereal crops in the world, ensuring food security and promoting economic development for many countries. The global need for wheat is expected to rise with the increasing world population (Tian *et al.*, 2021); this presents a significant challenge as the world needs to implement sustainable food production strategies, particularly under the pressure of current climate uncertainties (Chandio *et al.*, 2022; Yao *et al.*, 2023). Many strategies have been developed to meet the increasing demand for wheat, such as the development of climate-smart crops and disease-resistant varieties (Silva *et al.*, 2023), the adoption of innovative mixed cropping systems based on wheat (Tamburini *et al.*, 2020; Zhao *et al.*, 2020), and best farming practices including rational fertilization and use of bio-inoculant technologies (Zambrano-Mendoza *et al*., 2021; Benmrid *et al*., 2023; Silva *et al.*, 2023).

The potential role of microbial biotechnology through the application of microbial biofertilizers has gained global interest. Over the last two decades, basic and applied research on plant growth-promoting microorganisms (PGPM) including phosphate (P) solubilizers, has increasingly become a priority among private and public agricultural research institutions (Bargaz *et al.*, 2018). The use of microbial biofertilizers based on PGPM is often recommended to farmers as an environmentally friendly farming practice to improve crop yields (Vassilev *et al.*, 2015; Umesha *et al.*, 2018). This is due to their agro-ecological significance, economic benefit, and potential to drive the commercialization of biofertilizers [e.g. P solubilizing, N fixing (NF), etc.] that have gained the acceptance of farmers due to the success of several products in the market (Kamath *et al.*, 2023).

Among PGPM, plant growth-promoting fungi (PGPF) have proven beneficial to the growth and yield of crop plants under normal and stressed conditions (Hossain *et al.*, 2017). While bacterial plant growth promotion abilities have been extensively studied (Baris *et al.*, 2014; Nawaz *et al.*, 2020; Khourchi *et al.*, 2022), fungi are equally critical yet less explored, particularly in nutrient solubilization and acquisition by the plants. This underscores the need for more fungi-focused studies, as demonstrated in this work. Fungi positively affect seed germination, root morphogenesis, shoot growth, photosynthetic efficiency, and flowering (Tarroum *et al.*, 2022). PGPF can provide direct (e.g. biosynthesis of phytohormones and siderophores, solubilization/mineralization of nutrients) and/or indirect effects (e.g. repression of phytopathogens, defense response stimulation, tolerance to abiotic stresses functions for plant growth promotion) (Hossain *et al.*, 2017; Anwar *et al.*, 2021). Although both individual isolates and consortia of fungal species were reported to positively impact cereals (Hyakumachi *et al.*, 1994; Harman *et al.*, 2004; Gujar *et al.*, 2013), inoculation using fungal consortia (FC) proved to be more efficient, mainly due to their functional diversity and synergy resulting in enhanced PGP traits (Crossay *et al.*, 2019; Parvin *et al.*, 2020; Hett *et al.*, 2022).

A major environmental constraint of plant growth is limited P availability in many agricultural soils due to specific P retention phenomena, mainly adsorption, precipitation, and immobilization. Insoluble P forms such as rock P (RP) need to be solubilized and/or mineralized to facilitate plant uptake, an action that can be performed by PGPM (Aallam *et al.*, 2022; Elhaissoufi *et al.*, 2024), including PGPF (Xiao *et al.*, 2008; Klaic *et al.*, 2017; Zhang *et al.*, 2023). Wheat plants inoculated with a single P-solubilizing fungus increased available P by 135% and the P content in wheat plants by 225% compared with uninoculated plants (Singh and Reddy, 2011). Similarly, a mixture of phosphate-solubilizing fungi (PSF) (*Glomus constrictum*, *Aspergillus niger*, and *Penicillium citrinum*) positively enhanced P content and growth of wheat (Omar, 1997). Although significant progress has been made towards utilizing either individual species or FC-based approaches for PGP and P acquisition, findings are scarce in respect to understanding the wheat rhizoplane-associated fungal diversity and their functional PGP potential as niche-constructed FC.

A noticeable reduction in microbial diversity exists in the rhizoplane (the closest niche to the root) compared with the rhizosphere and bulk soil. The microbial communities are notably affected by the rhizo-compartment niches as there is increased selection for microbes with beneficial plant traits in the rhizoplane niche (Lang *et al.*, 2019; Park *et al.*, 2021). The niche concept succinctly captures the specific ecological role and interactions of organisms within a defined habitat (e.g. rhizoplane) (Kivlin *et al.*, 2021). The choice of rhizoplane niche aligns with the nuanced ecological functions that the present study explores across distant locations. Of particular interest are the functions related to P dynamics in the root–soil interface of roots (York *et al.*, 2016), where the rhizoplane fungal community can operate significantly to improve root development, P acquisition, and plant growth. Bioprospection of agroecological niches, such as the crop rhizoplane, enables the identification of individual species or a mixture of beneficial microbes for desired functions. However, optimal PGP functions can only be achieved when applied microbial consortia are able to co-exist and align well with the functional and ecological characteristics of the native microbiome, enhancing their establishment and efficacy. Synthetic microbial consortia are developed to generate defined communities with specific activities and reduced complexity compared with natural microbial communities that are difficult to produce and harbor unknown functions (Grosskopf and Soyer, 2014). Additionally, they offer advantages over single strain inoculants, which are constrained by low resistance and resilience to environmental changes and competition from the indigenous microbiome (Lindemann *et al.*, 2016). This concept of replicating the structure and functions of natural communities is of particular interest for biotechnological applications such as adapted crop inoculants for large-scale production to meet the growing global food demand (Grosskopf and Soyer, 2014; Sharma *et al.*, 2023). The production of fine-tuned synthetic FC from wheat rhizoplane-associated fungal communities is likely to have a significant impact on the nutrient uptake and growth of this important staple food. For instance, fungal isolation efforts based on P solubilization (PS) ability may select species with high PS capacity while excluding other co-existing species exhibiting key ecological functions within the natural ecosystem. Therefore, it is necessary to adopt a multi-PGP traits method based on co-existing microbes with diversified PGP functions to design effective consortia with a more holistic representation of the functional community within a specific niche, while conserving intra-PGP diversity through a niche-conserved strategy. Hence, culture-dependent and culture-independent methods should be coupled to generate complementary data on functional and taxonomic levels, to be considered for the construction strategy for the niche-conserved consortia. This study is the first of its kind to investigate the diversity and functions of rhizoplane-associated fungal communities specifically of wheat grown across seven agricultural regions in Morocco, with the aim of constructing rhizoplane FC based on a novel FC-oriented methodology (niche-conserved approach) that considers preserving the intra-community diversity features of the native fungal community. A fusion of culture-dependent and metataxonomic-based approaches considering ecological principles was adopted to build an understanding of microbial interactions around plant roots with a focus on functional traits related to PS and plant growth.

## Materials and methods

### Wheat plant sampling and extraction of rhizoplane soils

**Fig 1.**
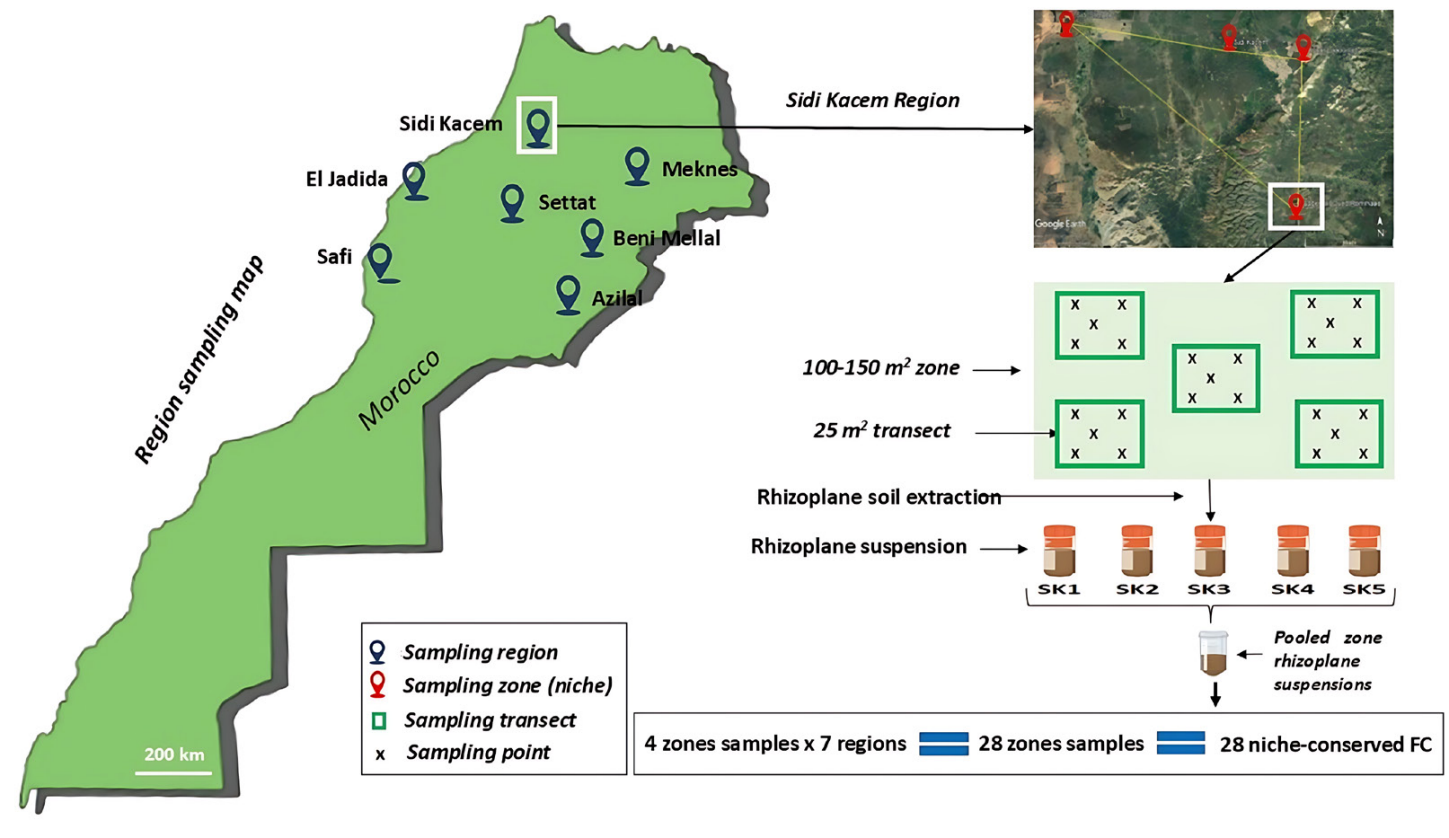
Sampling map of wheat plants and rhizoplane soils across seven different agricultural regions in Morocco. Rhizoplane samples were collected from seven agricultural region in Morocco (Sidi Kacem, Meknes, Beni Mellal, El Jadida, Settat, Azilal, and Safi). For each region, four zones (niches) were targeted and, from each zone, five plants sampled from five points along the 25 m^2^ transect were collected then pooled into one representative zone sample. In total, 28 zone samples were generated to give 28 FC. FC, fungal consortia; zone, niche. Map generated with Google map https://www.google.com/maps.

Wheat plants at the heading stage were sampled from 28 farm sites across seven wheat-growing regions in Morocco, namely Sidi Kacem, Meknes, Beni Mellal, El Jadida, Settat, Azilal, and Safi (Fig. 1). The sampling strategy encompassed sites with contrasting soil types, weather patterns, and cropping regimes using a standardized sampling protocol.

Four field zones (niches) were sampled within each region. The sampling of each field zone covered 100–150 m^2^ and consisted of five 25 m^2^ transects with five sampling points each. For each sampling point, five plants as well as 1 kg of bulk soil were sampled using an auger. The samples for each transect were then pooled and considered as a single replicate. In this way, five replicates of plant roots and bulk soil samples were created for each zone (Fig. 1). Samples (plants and soils) were collected in sterile plastic bags, immediately transported, and kept at 4 °C for no longer than 5 d prior to the rhizosphere-releasing and rhizoplane extraction process. Root systems were vigorously shaken to release the rhizosphere soil from the root system. Subsequently, 10 g of wheat roots were cut into segments of 4–6 cm in length, transferred to 50 ml tubes containing 25 ml of sterile distilled water, and rhizoplane soil was extracted from the root surface by vortexing and shaking (2 min/200 rpm/5 °C) (Wieland *et al.*, 2001; Lang *et al.*, 2019). The obtained rhizoplane suspensions were centrifuged (5300 *g*, 15 min, 4 °C) twice and the pellets (rhizoplane soils) were utilized for fungal culture isolation before they were suspended in glycerol (37%) and conserved at –20 °C for future experiments including DNA extraction for internal transcribed spacer (ITS) amplicon sequencing.

### Fungal isolation from wheat rhizoplane soils

**Fig 2.**
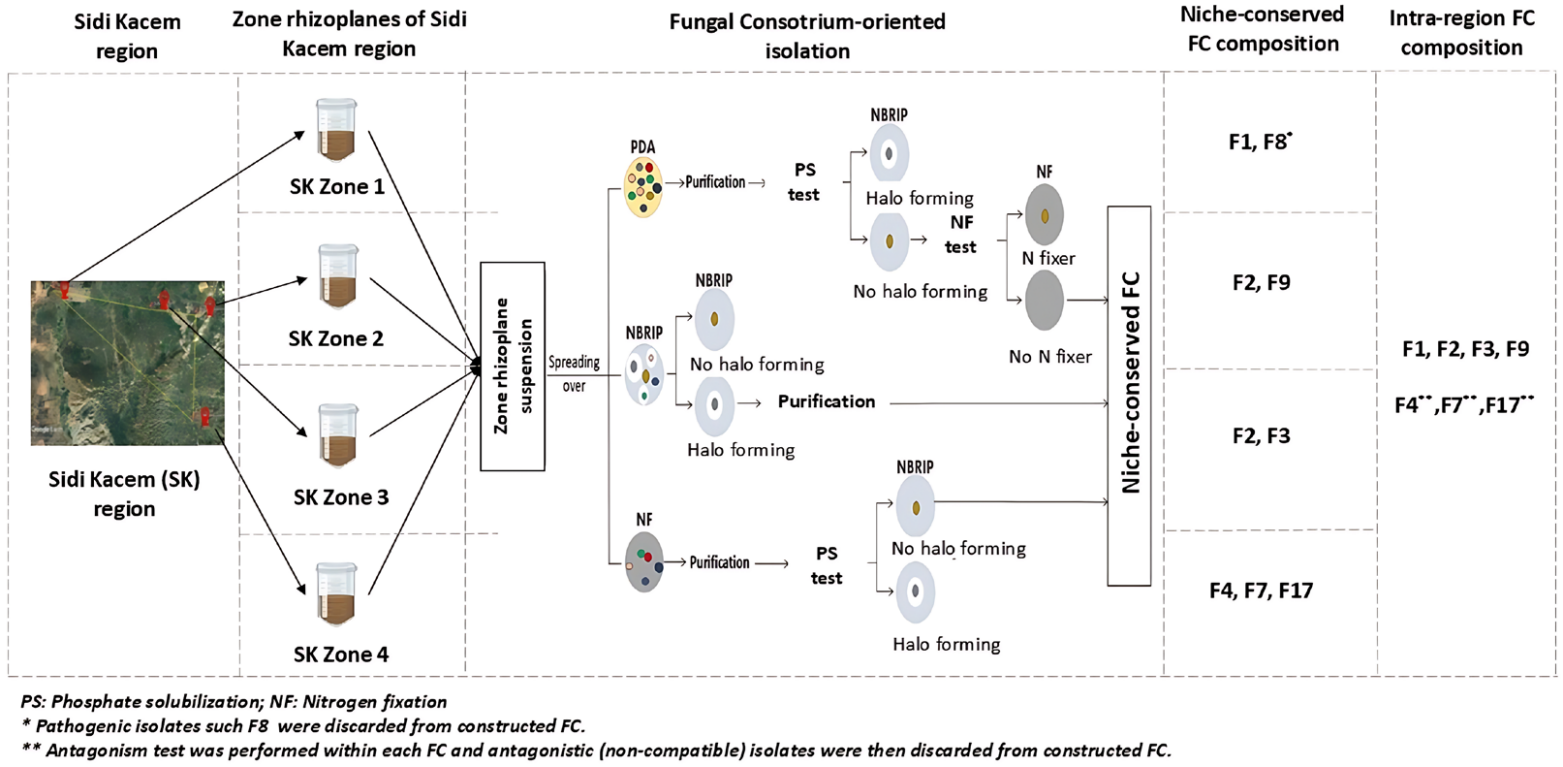
Design workflow of niche-conserved, intra-region, and inter-region FC using Sidi Kacem region as a model example. Each zone’s rhizoplane suspension underwent the FC-oriented isolation approach, generating a unique FC for each zone. Within each region, fungal isolates from the four niche-conserved (zone-specific) FC were combined to create an intra-region FC, representative of the given region. Subsequently, all fungal isolates across regions were pooled to form a general FC encompassing all studied regions, referred to as the inter-region FC. Pathogenicity and antagonism tests were performed across all species within the constructed FC to ensure they were non-pathogenic and synergistic.

Based on the standardized sampling procedure described above, replicates of the rhizoplane soils of each zone were pooled to generate one composite sample representing the niche’s natural microbial community (originating from each sampled zone). The FC-oriented isolation approach involved three culture media, namely potato dextrose agar (PDA), National Botanical Research Institute’s phosphate [NBRIP; 10 g l^–1^d-glucose, 5 g l^–1^ Ca_3_(PO)_2_, 5 g l^–1^ MgCl_2_·6H_2_O, 0.25 g l^–1^ MgSO_4_·7H_2_O, 0.2 g l^–1^ KCl, 0.1 g l^–1^ (NH_4_)_2_SO_4_, 15 g l^–1^ agar, pH 7], and nitrogen-free medium (10 g l^–1^d-glucose, 1.2 g l^–1^ KH_2_PO_4_, 0.8 g l^–1^ K_2_HPO_4_, 0.2 g l^–1^ MgSO_4_·7H_2_O, 0.2 g l^–1^ NaCl, 0.02 g l^–1^ CaCl_2_, 0.002 g l^–1^ FeSO_4_, 2 ml l^–1^ metal solution, 15 g l^–1^ agar, pH 7) (Fig. 2). PDA is a non-selective medium for fungi, whereas NBRIP and N-free media allow the isolation of PS and NF species only. In addition, a broad-spectrum antibiotic was added to prevent bacterial growth (streptomycin ~0.1 g l^–1^).

**Fig 3.**
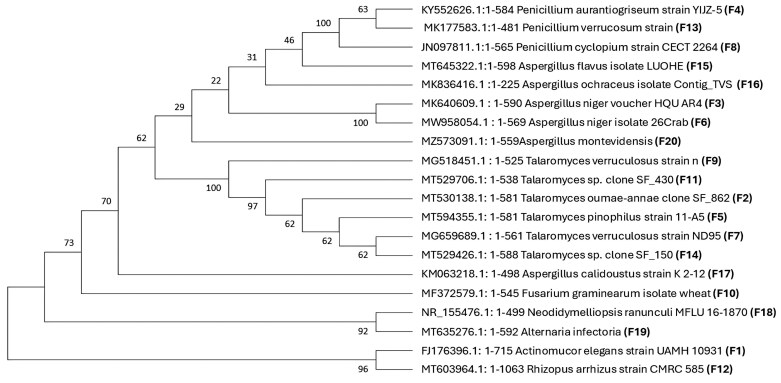
Phylogenetic tree of the fungal isolates and accession numbers according to the Neighbor–Joining method. F_1_–F_20_ are codes that correspond to each fungal species.

Rhizoplane soil suspensions (1 ml) were aliquoted from each pooled niche sample (28 rhizoplane samples), and serial dilutions (10^–1^–10^–6^) were prepared to be spread (100 µl each) simultaneously onto the three culture media. For the first isolation track (PDA medium), plates were incubated at 30 °C for 120 h and isolates that were morphologically different were re-plated five times before they were purified. The second track (NBRIP medium) consisted of capturing isolates that are exclusively PSF based on the development of solubilization halos around the colonies. Subsequently, PSF isolates were tested on N-free medium to exclude those that were developed. The same procedure was performed for the isolation of NF fungi, in the third track, that were tested for their inability to solubilize P (Fig. 3). Glycerol culture suspensions were prepared for all isolated fungi.

### Molecular identification of individual fungal isolates: genomic DNA extraction and ITS region gene sequencing

Fungal isolates were grown in PDA liquid medium for 72 h, then pelleted suspensions were collected and a bead-based homogenizer (Tissue Lyser II, QIAGEN) was used for fungal cell lysis [1 min at 30 Hz (~1800 oscillations min^–1^)]. Genomic DNA was extracted from homogenized pelleted fungal suspensions (1.5 ml) using the DNeasy® PowerSoil® Kit (QIAGEN, Hilden Germany), according to the manufacturer’s instructions (Matsuoka *et al.*, 2022). DNA extracts from all rhizoplane samples were visualized by agarose gel electrophoresis (0.8%), and spectrophotometrically quantified using a NanoDrop Microvolume spectrophotometer. PCR amplifications of the ITS region were performed using primers ITS1-F (5′-TCCGTAGGTGAACCTGCGG-3′) and 5.8S (5′-CGCTGCGTTCTTCATCG-3′). Each PCR was carried out in a 25 μl reaction volume: 12.5 μl of DreamTaq Green PCR Master Mix (2×) (Thermo Fisher Scientific, Wilmington, DE, USA), 1 μM of each primer, and 5 μl of DNA (5–10 ng). PCR amplifications were performed on a C1000 Touch Thermal Cycler (Bio-Rad Laboratories, Hercules, CA, USA) with an initial denaturation at 95 °C (3 min) followed by 35 cycles at 95 °C (30 s), 60 °C (30 s), and 72 °C (1 min), and a final elongation step at 72 °C (5 min). Amplification reactions were visualized by agarose gel electrophoresis (1.5%) before they were sent to Eurofins Genomics Germany for purification and Sanger sequencing. Consensus sequences, generated through the DNA Subway platform, were then subjected to the GenBank BLAST function on the NCBI website. The phylogenetic tree was conducted in MEGA11 (Tamura *et al.*, 2021) using the Neighbor–Joining method (Saitou and Nei, 1987). The percentage of replicate trees in which the associated taxa clustered together in the bootstrap test (1000 replicates) were indicated next to the branches (Felsenstein, 1985). The evolutionary distances were computed using the p-distance method (Nei and Kumar, 2000) (Fig. 3).

### Rhizoplane soil DNA extraction and ITS amplicon sequencing

Total soil DNA was extracted from rhizoplane soil (~0.25 g) using the DNeasy PowerSoil Pro kit (Qiagen, Venlo, The Netherlands) and stored at –80 °C. Extractions were performed according to the manufacturer’s instructions but with the use of the MP Biomedicals FastPrep-24 machine twice for 30 s at an amplitude 5.5 m s^–1^. DNA purity and concentrations were established by NanoDrop spectrophotometry (Thermo Fisher Scientific) and a Qubit 2.0 Fluorimeter using the dsDNA BR assay kit (Thermo Fisher Scientific), respectively. DNA samples were sent to Novogene (UK) Company Limited (Milton, Cambridge, UK) and subjected to dual index paired-end (2×250bp) sequencing using the Illumina MiSeq platform. Amplicons spanning the ITS 1 region were produced using primers ITS2 (5′-GCTGCGTTCTTCATCGATGC-3′) and ITS5 (5′- GGAAGTAAAAGTCGTAACAAGG-3′).

#### Sequence processing and analysis

Raw sequence reads were processed using the pipeline employed at Novogene. Briefly, paired-end reads were demultiplexed according to sample barcodes and filtered using the cutadapt v.3.3 (Martin, 2011) function in Python v3.6.13. The resulting reads were merged using the FLASH (Fast Length Adjustment of Short reads) v.1.2.11 (Magoč and Salzberg, 2011) plugin implemented in Geneious. Merged reads were quality filtered using fastp v.0.23.1 software (Chen *et al.*, 2018) and chimeras were removed using the UCHIME algorithm (Edgar *et al.*, 2011).

The resulting high-quality merged sequence reads were then imported into QIIME 2 2019.7 (Bolyen *et al.*, 2019) using the q2-demux plugin for demultiplexing and quality filtering followed by denoising using DADA2 (Callahan *et al.*, 2016) (via q2-dada2) where sequences were processed into amplicon sequence variants (ASVs) using default parameters. ASVs with <10 observations in the dataset were removed via q2-filter-features and filter-seqs. Fungal taxonomy was assigned using the q2-feature-classifier (Bokulich *et al.*, 2018) classify-consensus-vsearch (Rognes *et al.*, 2016) against the UNITE database (version 9.0) (Nilsson *et al.*, 2019). Unassigned ASVs were removed and the resulting ASVs were aligned with mafft (Katoh *et al.*, 2002) and used to build a phylogenetic tree with fasttree (Price *et al.*, 2010) using the q2-align-to-tree-mafft-fasttree function via q2-phylogeny.

To identify whether any isolates corresponded to major ASVs within the ITS gene amplicon dataset, the ITS forward and reverse sequences and corresponding taxonomy (kingdom to strain levels) from the cultured fungal isolates (*n*=20) were used to create Qiime 2 taxonomy and ASV files via the q2-tools import command as previously described in Reid *et al.* (2021). Fungal isolate taxonomy was assigned as described above but using the taxonomy and ASV QIIME 2 files as the reference database. The resulting ASV tables and taxonomy assignments (from the UNITE classification and the isolate classification), and phylogenetic tree were exported for downstream analysis.

Microbial community analysis was performed in R v4.2.2 (R Core Team, 2022) using the Phyloseq (v1.42.0) (McMurdie and Holmes, 2013) package. Data were rarefied to the minimum read count (UNITE: 24 766, Isolate: 7873) (Supplementary Fig. S1) by random subsampling using the rarefy_even_depth function. ASVs no longer present in any sample after random subsampling were removed (UNITE: 111 ASVs, Isolate: 2 ASVs). Differences in observed ASV richness (R package microbiome) (Lahti and Shetty, 2012–2019) and Faith’s phylogenetic diversity (Faith, 1992) (R package picante v1.8.2) (Kembel *et al.*, 2010) between sampling regions were investigated by one-factor ANOVA tests followed by post-hoc comparisons using Tukey’s honest significant differences test (HSD.test function in package agricolae) (de Mendiburu, 2021) and visualized using ggplot2 (Wickham, 2016) according to code from Simonin *et al.* (2020). The Shapiro–Wilk test was applied to assess data normality, while Levene’s test was used to check the homogeneity of variances. To compare fungal kingdom and genus abundances in both datasets and across zones, ASV numbers were normalized by the variance stabilizing transformation method using the package DESeq2 v1.36.0 (Love *et al.*, 2014). The resulting normalized ASV tables were used to plot the relative abundance of ASVs at kingdom level for all ASVs (4393 ASVs) and at genus level for the 231 ASVs that were identified as fungal isolates in the UNITE dataset and the Isolate dataset, using the metagMisc package (Mikryukov, 2022) for data manipulation and ggplot2 (Wickham, 2016) for data visualization.

### Functional characterization of fungal isolates

#### Phosphate solubilization and production of organic acids

To determine the *in vitro* PS potential of fungal species, a 1 ml aliquot of each fungal inoculum (~10^6^ CFU ml^–1^) was incubated in NBRIP liquid medium for 10 d at 30 °C and under gentle shaking (180 rpm). Each suspension was centrifugated (10 000 *g* for 10 min) and the supernatant was used to quantify available P according to Murphy and Riley (1962) using a microplate reading spectrophotometer (Fluostar Omega) and to measure NBRIP medium pH using the pH meter Mettler-Toledo (Switzerland). The same supernatants were filter sterilized (using a 0.2 μm filter) and used to quantify organic acids (OAs) by HPLC as described by Santos-Torres *et al.* (2021). The filtered samples were stored at –20 °C before injection into a BioRad HPX-87H column at 65 °C, with a mobile phase (sulfuric acid) flow rate of 0.5 ml min^–1^. The acetic, formic, glycolic, galacturonic, glucuronic, fumaric maleic, succinic, pyruvic, citric, malic, oxalic, lactic, and gluconic acids were used as analytical standard acids.

#### Acid and alkaline phosphatase activities

The cell-free supernatant of 10-day-old inoculated NBRIP medium was assayed for acid (AP_ase_) and alkaline (AlkP_ase_) phosphatase activities according to Tabatabai and Bremner (1969). An aliquot (~125 μl) of fungal cell-free supernatant was added to acetate buffer (pH 5.6, 0.2 M; acid phosphatase) or universal modified buffer (pH 11, 0.2 M; alkaline phosphatase) and disodium *p*-nitrophenyl phosphate (*p*-NPP, 10 mM). The homogenate was vortexed and incubated at 37 °C for 1 h. After incubation, the enzymatic reaction was stopped by adding 500 μl of NaOH (0.5 M) and 125 μl of CaCl_2_ (0.5 M). After centrifugation (10 000 *g*, 5 min, 4 °C), the amount of *p*-nitrophenol released into the medium was spectrophotometrically determined at 405 nm and both phosphatase activities were expressed as the amount of enzyme required to release 1 nmol of *p*NP ml^–1^ h^–1^ from *p*NPP.

#### Potassium solubilization ability and zinc solubilization index

Fungal isolates were tested for their potassium solubilization (KS) and zinc solubilization (ZS) capacities on Aleksandrov (AM) and Basal (BM) agar medium, respectively. Aliquots of 10 ml of fresh fungal suspensions were deposited on AM and BM agar and incubated for 7 d, at 30 °C, with three replications. The detection of a clear orange zone in AM indicates KS according to Prajapati and Modi (2012). The ZS index was measured as the total diameter of both the colony and the halo divided by the colony diameter (Saravanan *et al.*, 2003).

### Evaluation of individual fungal inoculation on seed germination and 20-day-old seedling growth of wheat

Fungal isolates were tested for their seedling growth-promoting trait, but also for stimulating or inhibiting seed germination. Individual fungal inocula were prepared in potato dextrose broth (PDB) liquid medium and incubated at 30 °C for 5 d. Wheat seeds (*Triticum durum*, variety Karim) were surface-disinfected with sodium hypochlorite (6%, 1 min) and ethanol (96%, 1 min) before they were washed thoroughly with sterile distilled water several times to remove excess chloride. Disinfected seeds were mixed with 10 ml of fungal inoculum (~10^6^ spores ml^–1^) for 2 h with gentle shaking (~100 rpm) and were germinated in Petri dishes for 2 d at 26 °C (day), 23 °C night. Germination rates (%) were determined and the germinated seeds were transferred into a 1 liter black plastic pot filed with sterile perlite, enriched with 20 ml of Hoagland nutrient solution [macronutrients (g l^–1^): Ca(NO_3_)_2_, 1.18; MgSO_4_·7H_2_O, 0.246; KNO_3_, 0.505; micronutrients (mg l^–1^): H_3_BO_3_, 2.886; MnCl_2_·4H_2_O, 1.81; ZnSO_4_·7H_2_O, 0.22; CuSO_4_·7H_2_O, 0.08; H_2_MoO_4_·H_2_O, 0.025 g l^–1^; and Fe EDTA, 0.5 ml l^–1^], and incubated in a plant growth chamber under controlled conditions (28 °C, 70% humidity, 16/8 h photoperiod, and an illumination intensity of 240 mmol m^–2^ s^–1^). Twenty days after transplantation, seedlings were harvested and assessed in terms of biomass and height of shoots. Root morphological traits (total root length ‘RL’, root surface area ‘RSA’, root average diameter ‘RD’, and root volume ‘RV’) were measured using the automated image analysis software WinRhizo (Regent Instruments Inc., Quebec City, Canada).

### Antagonism test and construction of fungal consortia

The concept behind the niche-conserved FC approach aimed at constructing 28 FC corresponding to each sample zone (4 zones×7 regions). Additionally, seven intra-region FC were designed, incorporating fungal isolates from the four niches within each region. Lastly, an inter-region FC was designed by combining all seven intra-region FC. To retain only non-plant pathogen and compatible species, three phytopathogenic fungal species (*Penicillium cyclopium* strain CECT 2264 ‘F_8_’, *Fusarium graminearum* isolate wheat ‘F_10_’, and *Alternaria infectoria* ‘F_19_’) were discarded based on the literature and the inhibition gemination assay described above. The next step was to keep only synergistic non-antagonistic species within each FC. For this, antagonism tests were conducted crossing all species within niche-conserved constructed FC. This test was done on PDA medium (5 d at 30 °C) by linearly streaking an isolate against a second isolate aligned perpendicularly. The growth of intercrossed isolates indicated absence of antagonism. Upon excluding antagonized species from the FC composition, 13 FC were identified to comprise only one species, prompting their removal from consideration. Eventually, 23 FC comprising 15 niche-conserved (or zonal FC), seven intra-region (FC_R1_–FC_R7_), and one inter-region ‘global’ (FC_G_) FC were constructed (Supplementary Table S1) and tested for their PGP ability and P acquisition of wheat growth under low available P conditions.

### Evaluation of inoculation of fungal consortia on agro-physiological performance of wheat (vegetative and heading stages) under rock P supply

The PGP potential of the 23 refined constructed FC was tested on 30- (vegetative stage) and 70-day-old (reproductive stage) wheat plants grown under controlled conditions (28 °C, 70% humidity, 16/8 h photoperiod, and an illumination intensity of 240 mmol m^–2^ s^–1^). Their effect was also tested in terms of plant P uptake under limiting P conditions by using RP as an insoluble P source. Disinfected wheat seeds and inocula were prepared following the same methodology indicated above. Individual fungal inocula were prepared for each isolate, and fungal cultures were adjusted to 10^6^ spores ml^–1^, then FC were prepared by mixing individual isolates of each FC in equal ratios. Disinfected seeds were immersed in FC inocula for 2 h with gentle shaking (~100 rpm) before they were sown in sterile pots (9.5 cm in diameter and 30 cm in height) filled with a sterilized mixture of soil (native of Ben Guerir city with 35% clay and a pH of 8.2) and sand for better aeration. The mixture (soil:sand, 3:1, v:v) was characterized to be a low-P substrate (P Olsen, 3.5 ppm; pH, 8,17). Prior to filling pots, the substrate was amended with RP based on wheat P requirements equivalent to 300 kg ha^–1^. Three uninoculated control treatments were used: amended with RP, ‘RP’; amended with ortho-phosphate, ‘Ortho-P’ (78 kg P_2_O_5_); and uninoculated and non-amended treatment, ‘P_0_’. The pots were watered twice a week with 10 ml of Hoagland nutrient solution lacking P while maintaining soil field water holding capacity at 70%. The experimental design was a randomized complete block of five replicates per treatment with each replicate consisting of a pot with two wheat plants. Plants were harvested at the vegetative and reproductive growth stages, then above- and below-ground growth parameters were assessed.

#### Measurement of plant growth and nutrient uptake

For both 30- and 70-day-old plants, plant height and biomass of shoots and roots were determined. Shoot dry weight (SDW) and root dry weight (RDW) were determined after drying the fresh biomass for 2 d at 80 °C. Samples of SDW were finely ground using a ball mill (Grinder, RETSCH, MM 400; 30 s at 5.5 m s^–1^) and the finely ground subsamples were used for shoot nutrient content. Samples were digested using nitric acid and analyzed for P, K, and N contents using inductively coupled plasma optical emission spectrometry (Agilent 5110 ICP-OES, USA).

#### Measurement of chlorophyll content and leaf area

The photosynthetic efficiency of 30- and 70-day-old plants was assessed by measuring the chlorophyll content index (CCI) using a chlorophyll meter (CCM-200 plus). Leaf area was measured using a LI-3100C Area Meter. Individual leaves were deposited on the transparent conveyor chain and the scanned cumulative surface areas of plant leaves were measured.

#### Measurement of root morphological traits

Root morphological traits including (root length) RL, (root surface area) RSA, and (root volume) RV were measured using the automated image analysis software WinRhizo (Regent Instruments Inc., Quebec City, Canada).

#### Determination of the rhizosphere-available P

The rhizosphere soils were collected, dried, sieved, and used to determine the available P fraction according to Murphy and Riley (1962). Aliquots of 1 g of dried rhizosphere soils was added to 10 ml of sodium bicarbonate (NaHCO_3_ 0.5 M; pH 8.5), shaken for 30 min at 150 rpm, and passed twice through Whatman filter paper (40 μm). Rhizosphere supernatants (1 ml) were used to measure the available P spectrophotometrically at 880 nm with reference to a standard KH_2_PO_4_.

### Statistical analysis

The results were analyzed by one-way ANOVA using the IBM^®^ SPSS^®^ software 27.0.1 package for Windows. The data were tested for normal distribution and homogeneity of variances to ensure the validity of the analysis. Significant differences between means were compared using Tukey’s test to determine the significant difference between the means of the treatments at the *P*<0.05 significance level. The results were expressed as the mean ±SD. Pearson correlation coefficients were calculated to study the correlation between the concentration of solubilized P, organic acid concentration, and pH.

## Results

### Identification of individual fungal isolates and abundance within the total amplicon fungal community

Using the FC-oriented isolation approach, 20 distinct fungal species (F_1_–F_20_) were isolated (Fig. 3). As the approach involved three distinct culture media, the isolated fungi were classified based on the specific isolation medium as follows: 11 fungi were classified as PGPF, cultivated on PDA; five fungi as PSF, in NBRIP medium, and four displayed NF capabilities. The fungal isolates were also presented according to their region of isolation. The highest functional fungal diversity was observed in the regions of Sidi Kacem and Safi, with nine species isolated from each, followed by Settat with eight species. Seven species were isolated from Meknes, El Jadida, and Beni Mellal, In Azilal, six distinct fungal species were isolated. Some fungi were present in more than one region (Supplementary Table S1).

Through the examination of the ITS sequences, the fungal isolates featured a variety of genera, prominently including *Aspergillus*, represented by five distinct genera, *Penicillium* (four species), *Talaromyces* (three species), and one representative species from *Rhizopus*, *Neodidymelliopsis*, *Alternaria*, and *Fusarium* (Fig. 3).

**Fig 4.**
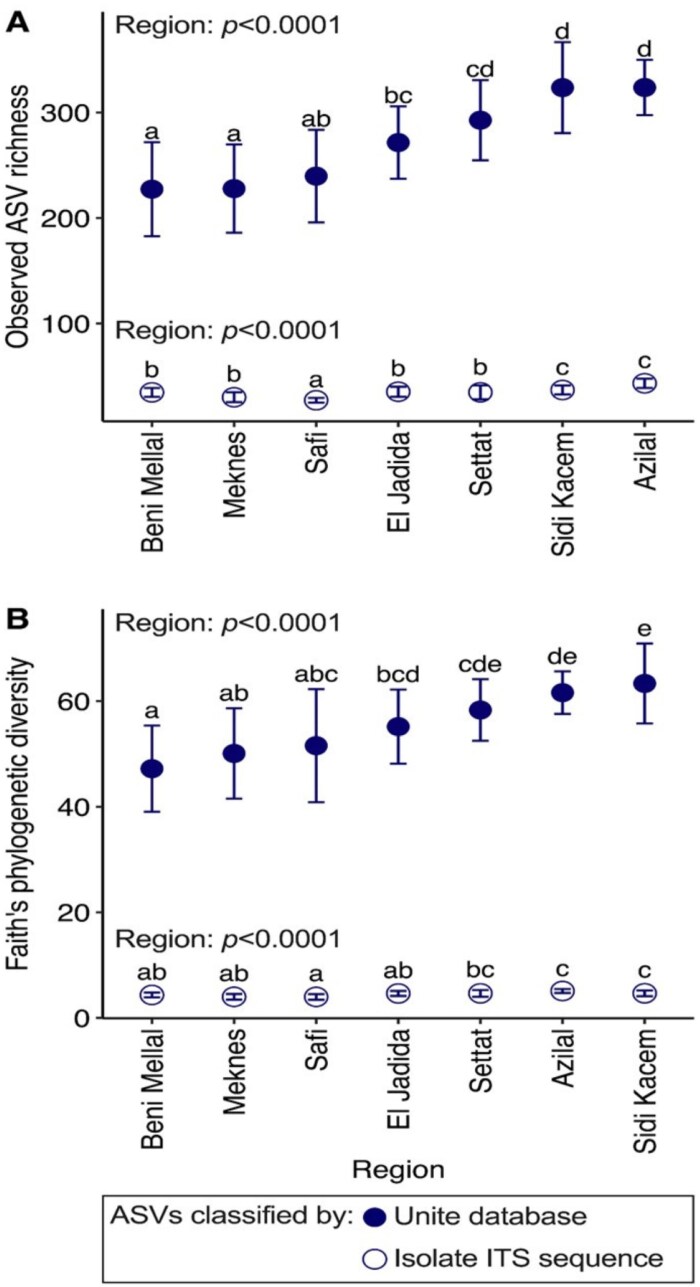
Influence of the seven agricultural regions on rhizoplane fungal diversity. (A) Observed amplicon sequence variant (ASV) richness and (B) Faith’s phylogenetic diversity in wheat rhizoplane samples from seven regions for the total fungal amplicon (ASVs classified by UNITE) and isolate (ASVs classified by isolate ITS sequences) community. ASV abundances were rarefied, yielding 4457 ASVs, 230 of which were identified as isolated fungal strains. Regions on the *x*-axis are ordered from low to high total fungal diversity. The average and SDs are represented. The statistical differences between the seven regions were determined by one-factor ANOVA followed by a post-hoc Tukey test and are represented by different letters: two regions sharing the same letter are not statistically different (*P*>0.05).

**Fig 5.**
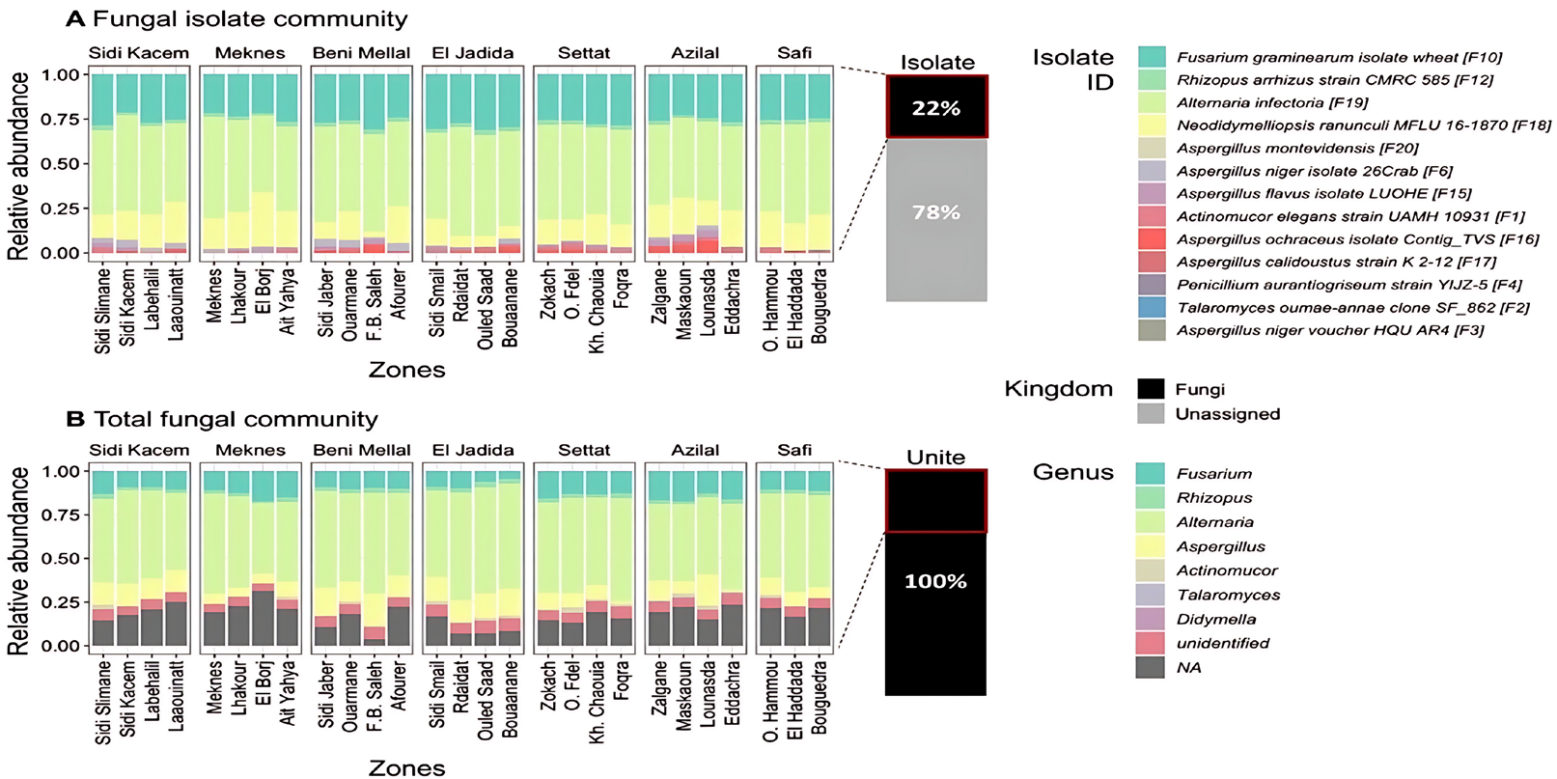
Taxonomic annotation of rhizoplane ASVs classified as culturable fungal isolates found in agricultural niches (zones) sampled from seven regions in Morocco. Bars represent relative abundance of amplicon sequence variants (ASVs) classified by (A) isolate ITS sequences and (B) the UNITE database, for each zone (*n=*3–5) in the seven sampled regions. The ASV abundances have been standardized by DESeq2 median of ratios, yielding 4393 ASVs from rhizoplane samples, 231 of which were also identified as the isolated fungal strains. The number of ASVs classified as each genus is listed in Supplementary Table S2.

Mapping of the fungal isolate ITS sequences to the culture-independent amplicon dataset revealed that the identified isolated fungal strains represented 5% (231 ASVs) of these ASVs. Wheat rhizoplane fungal richness and phylogenetic diversity differed among regions for total fungal community [richness: ANOVA (degrees of freedom=6) *F*=22.79, *P*<0.0001] (diversity: *F*=12.74, *P*<0.0001) and isolate fungal community (richness: *F*=21.83, *P*<0.0001) (diversity: *F*=11.41, *P*<0.0001) (Fig. 4). Sidi Kacem and Azilal regions had the highest fungal richness (total number of species) and phylogenetic diversity for both total fungal community and isolate presence compared with Meknes and Beni Mellal which had the lowest (Fig. 4). Within the total fungal community, fungal isolates comprised 22% of the total abundance, with the remaining 78% consisting of ASVs unassigned at kingdom level, whereas all ASVs classified by UNITE were identified as fungi (Fig. 5; Supplementary Fig. S1A). Notably, *Alternaria infectoria* (F_19_) (88 ASVs), *Neodidymelliopsis ranunculi* MFLU 16-1870 (F_18_) (44 ASVs), and *Fusarium graminearum* isolate wheat (F_10_) (42 ASVs) had the highest abundances across all zones (Fig. 5A; Supplementary Table S2). We further examined the 231 ASVs identified as fungal strains from the UNITE dataset (Supplementary Fig. S1B). Among these, *Alternaria* dominated with 88 ASVs, followed by 48 ASVs that were unclassified at the genus level (<NA>), and *Aspergillus* with 42 ASVs (Fig. 5B). Interestingly, two ASVs that were identified as *N. ranunculi* MFLU 16-1870 (F_18_) were classified as *Didymella*, a genus of the same family *Didymellaceae*, leaving 42 ASVs unassigned. Additionally, the ASV classified as *Penicillium aurantiogriseum* strain YIJZ-5 (F_4_) was classified as an *Aspergillus* species, also of the same family. When considering all genera in the dataset (311 in total), 76 ASVs were classified as *Penicillium*, suggesting that other *Penicillium* species were more prevalent. However, the genus *Neodidymelliopsis* was absent despite it being the third most abundant isolate (Supplementary Table S2).

### Functional characterization of fungal isolates

**Fig 6.**
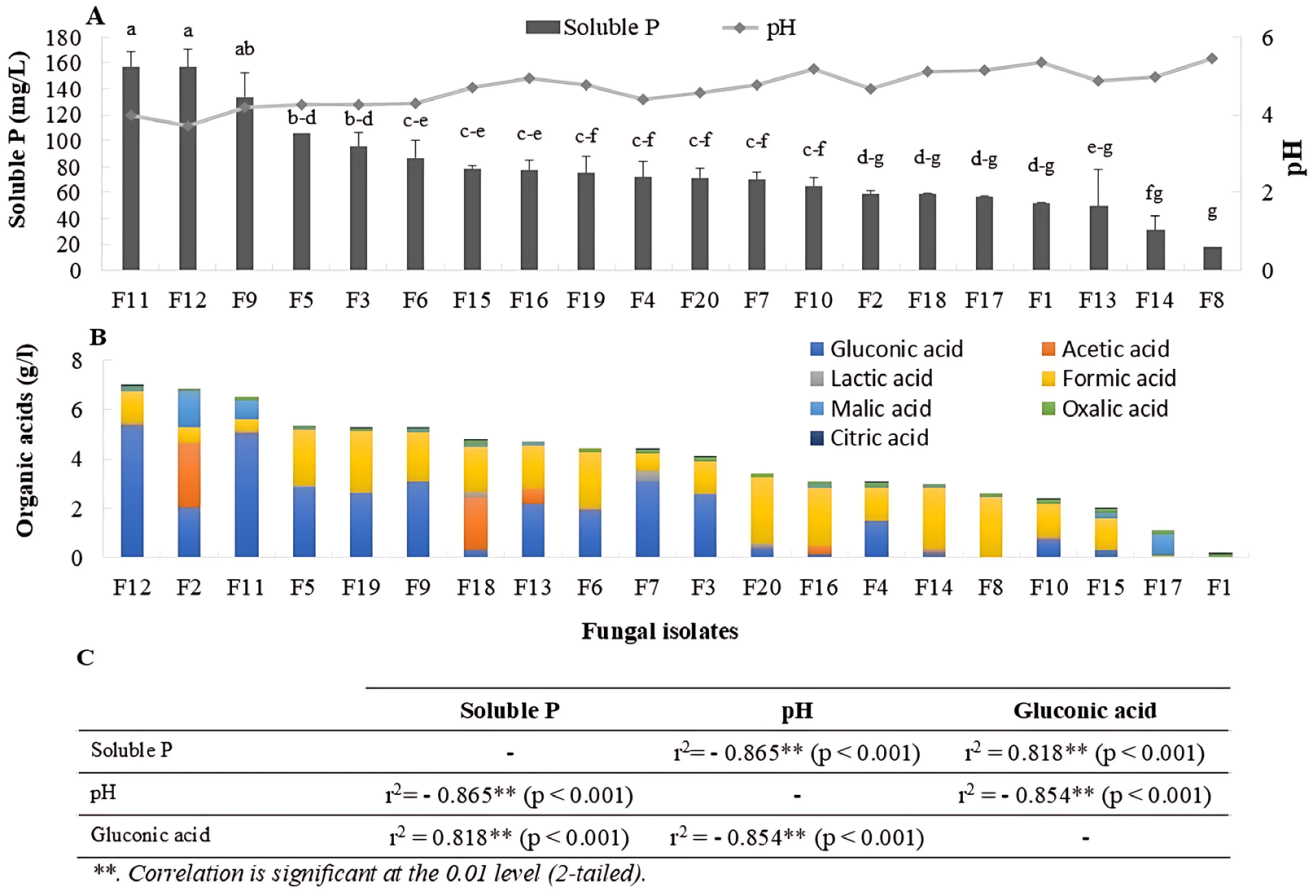
Soluble P concentrations (mg l^–1^) and pH (A), organic acid profiling (B) in NBRIP liquid medium supplemented with TCP and inoculated with individual fungal isolates (F_1_–F_20_), and (C) Pearson correlation between soluble P, pH, and gluconic acid concentration. The results are means ±SD (*n*=3). Bars are displayed in descending order from left to right. Different letters indicate values that are statistically different at *P*<0.05.

Twenty fungal isolates were tested for their PS capacity, OA production, phosphatase activities, ZS, and KS. In terms of PS, all isolates were able to dissociate the insoluble tri-calcium phosphate (TCP), generating available P concentrations ranging from 17.8 mg l^–1^ to 157.3 mg l^–1^. Isolate F_11_ (best match *Talaromyces* spp. Clone SF_430) and F_12_ (best match *Rhizopus arrhizus* strain CMRC 585) had the highest PS function (~157.30 mg l^–1^) (Fig. 6). Other isolates, particularly F_9_ (best match *Talaromyces verruculosus* strain n) and F_5_ (best match *Talaromyces pinophilus* strain 11-A5) were moderate P solubilizers (100–130 mg l^–1^), while the remaining isolates exhibited PS capacities ranged from 90 mg l^–1^ to 17.8 mg l^–1^. Concomitantly, these four fungal species significantly acidified the growth medium (pH 3.7–4.2) as compared with the low PS species with a pH ranging from 4.5 to 5.4 (Fig. 6A).

Medium acidification is likely to be due to OA production, with gluconic, acetic, lactic, formic, malic, oxalic, and citric acids being the most produced (Fig. 6B). Formic acid was produced (0.44 mg l^–1^ and 2.67 mg l^–1^) by all isolates, whereas gluconic acid, the OA most positively correlated with PS, was produced by 17 isolates, with the highest rates (5.05 mg l^–1^ and 5.37 mg l^–1^) by isolates F_11_ and F_12_. Gluconic acid concentration significantly correlated with soluble P concentration (*r*^2^=0.818; *P*<0.001), which was logically the opposite between pH and gluconic acid concentration (*r*^2^= –0.854; *P*<0.001) (Supplementary Table S4).

The ability of the fungal isolates to secrete both acid (AP_ase_) and alkaline (AlkP_ase_) phosphatase was assessed (Table 1). All isolates produced both phosphatase enzymes, ranging from 461.25 nmol ml^–1^ h^–1^ to 1361.25 nmol ml^–1^ h^–1^ for AP_ase_ and from 427.5 nmol ml^–1^ h^–1^ to 1781.25 nmol ml^–1^ h^–1^ for AlkP_ase_, with the average activity of AP_ase_ (871.12 nmol ml^–1^ h^–1^) being much higher than that of AlkP_ase_ (732.56 nmol ml^–1^ h^–1^), which indicated the optimum activity in acidic rather than alkaline conditions. Isolate F_16_ (best match *Aspergillus ochraceus* isolate Contig_TVS) had the highest AP_ase_ activity (1361.25 nmol ml^–1^ h^–1^), while the highest AlkP_ase_ activity (1781.25 nmol ml^–1^ h^–1^ ) was noted in isolate F_7_ (best match *T. verruculosus* strain ND95) (Table 1).

**Table 1.** *In vitro* PGP traits of the fungal isolates and wheat seedling growth parameters in response to inoculation with individual fungal isolates

	*In vitro* PGP traits				20-day-old *in planta* growth traits						
Isolate ID	AP_ase_ (nmol ml^–1^ h^–1^)	AlkP_ase_ (nmol ml^–1^ h^–1^)	KS	ZSI	Germination rate (%)	SH (cm)	SDW (g)	RL (cm)	RSA (cm^2^)	RD (mm)	RV (cm^3^)
T	–	–	–	–	86.7 a	13.8 efg	0.6 e–h	90.0 efg	12.3 a–d	0.4 de	0.1 c–f
F_1_	456.9 g	453.7 e	–	1.3 h	73.3 bcd	18.0 bc	0.6 d–h	116.2 abc	13.7 ab	0.4 b–e	0.1 bcd
F_2_	1023.2 cd	442.5 e	+	2.0 e	66.7 c–f	18.7 ab	0.7 c	127.2 a	14.8 a	0.4 a–e	0.2 ab
F_3_	540.7 fg	438.7 e	–	3.6 a	73.3 bcd	16.7 def	0.7 b	68.3 hi	9.8 d	0.4 a–e	0.1 ef
F_4_	522.6 fg	915.0 c	-	2.4 d	63.3 def	16.7 def	0.7 cde	104.7 c–f	12.1 a–d	0.4 b–e	0.2 b–e
F_5_	627.0 ef	963.7 c	–	2.0 e	63.3 def	18.0 bcd	0.5 ghi	65.4 hi	9.5 d	0.4 b–e	0.1 f
F_6_	763.7 e	2955.0 a	–	2.8 c	56.7 fg	14.2 d–g	0.5 ghi	91.0 efg	11.4 bcd	0.4 cde	0.1 ef
F_7_	1158.0 bc	1781.2 b	+	1.9 e	76.7 b–e	17.8 bcd	0.6 def	105.9 b–e	12.6 a–d	0.4 a–e	0.1 c–f
F_8_	592.8 fg	453.8 e	+	1.0 j	13.3 k	0.0 h	0.0 k	0.0 k	0.0 e	0.0 f	0.0 g
F_9_	628.1 ef	772.5 cd	–	2.0 e	70.0 cde	18.8 ab	0.8 a	59.0 ij	14.8 a	0.4 cde	0.2 a
F_10_	658.4 ef	442.5 e	+	1.3 h	30.0 i	12.8 fg	0.5 hi	94.4 d–g	11.8 a–d	0.4 b–e	0.1 def
F_11_	955.9 d	510.0 e	–	1.8 f	80.0 ab	19.7 a	0.8 a	124.4 ab	15.1 a	0.5 abc	0.2 abc
F_12_	632.0 ef	491.2 e	+	3.0 b	70.0 c–f	18.3 ab	0.8 a	104.0 c–f	14.7 ab	0.4 a–e	0.1 bcd
F_13_	1182.9 abc	427.5 e	–	1.5 g	63.3 def	17.3 cde	0.6 e–h	107.6 b–e	13.9 ab	0.4 e	0.1 c–f
F_14_	616.0 efg	461.2 e	+	–	83.3 a	18.2 bc	0.7 bc	119.7 abc	14.7 ab	0.4 a–e	0.2 bcd
F_15_	625.7 ef	480.0 e	–	1.0 j	63.3 def	15.2 d–g	0.6 f–i	45.3 j	10.3 cd	0.5 a	0.1 ef
F_16_	1234.0 ab	502.5 e	–	1.0 j	80.0 ab	16.8 de	0.7 bc	110.7 a–d	14.2 ab	0.5 a–d	0.2 abc
F_17_	1150.0 bc	465.0 e	–	1.1 ij	83.3 a	18.3 ab	0.7 bc	124.5 ab	14.5 ab	0.4 cde	0.1 bcd
F_18_	1328.7 a	465.0 e	+	–	60.0 ef	17.2 cde	0.7 de	86.0 fg	13.5 abc	0.5 abc	0.2 bcd
F_19_	1330.6 a	667.5 de	+	1.0 j	50.0 fgh	14.0 efg	0.3 j	78.2 gh	9.9 d	0.4 de	0.1 f
F_20_	1048.7 cd	562.5 de	–	1.2 i	66.7 c–f	17.8 cd	0.7 cd	95.3 d–g	14.3 ab	0.5 ab	0.1 bcd

AP_ase_, acid phosphatase activity; AlkP_ase_, alkaline phosphatase activity; KS, potassium solubilization activity; ZSI, zinc solubilization index; SH, shoot height; SW, shoot weight; RL, root length; RSA, root surface area; RD, root average diameter; RV, root volume; T, uninoculated control; +, presence of activity; –, absence of activity.

Values are means ±SD. Different letters indicate values that are statistically different at *P*<0.05.

In addition, PGP traits related to KS and ZS revealed functional differences among fungal species, with only eight isolates being able to solubilize K; among these was the high P-solubilizing isolate F_12_. For ZS, isolate F_3_ (best match *A.* niger voucher HQU AR4) as well as isolates F_12_ and F_11_ had the highest ZS index, with 3.7, 3, and 2.85, respectively (Table 1).

### Effect of fungal inoculation on seed germination and seedling growth parameters of wheat

Only four (F_8_, F_10_, F_19_, and F_6_) isolates appeared to reduce the germination rate as compared with uninoculated treatment (Table 1). As they also impaired seedling development (at 20 d old), these species were likely to be pathogenic, particularly isolate F_8_ (best match *P. cyclopium* strain CECT 2264), which completely inhibited seedling growth and root morphological parameters (Table 1). Meanwhile, isolates F_17_ (best match *Aspergillus calidoustus* strain K 2-12), and F_2_ (best match *Talaromyces oumae-annae* clone SF_862) significantly improved weight and height of 20-day-old wheat seedlings as compared with the uninoculated control. This positive effect was also noticed for root morphological parameters including RL, RSA, RD, and RV (Table 1).

### Antagonism and construction of fungal consortia

It was found that seed germination inhibition tests confirmed the phytopathogenicity of three fungal isolates F_8_, F_10_, and F_9_, and they were removed from the list to serve for constructing FC. Next, isolates within the same niche (zone) were tested for their biocompatibility based on antagonism tests, and the antagonistic fungal isolates were also removed and considered for FC construction (Table 1). Antagonism was rarely observed between isolates belonging to the same niche (zone); however, several fungi were removed from intra- and inter-region FC due to antagonism. Refining FC by retaining only compatible isolates generated 23 FC, including 15 niche-conserved FC (FC_2_, FC_3_, FC_4_, FC_6_, FC_8_, FC_9_, FC_13_, FC_14_, FC_15_, FC_17_, FC_18_, FC_21_, FC_22_, FC_26_, and FC_28_), seven intra-region FC (FC_R1_–FC_R7_), and one inter-region ‘global’ FC (FC_G_).

### Effect of fungal consortia inoculation on 30- (vegetative) and 70-day-old (reproductive) wheat plant growth with rock P supply

#### In situ *parameters*

The impact of FC on the CCI revealed a consistent improvement across two wheat plant growth stages. In the vegetative stage (30-day-old plants), FC_14_ and FC_17_ exhibited an increased CCI of 21.8% and 7.8%, respectively, over the Ortho-P treatment. Notably, such a positive effect was also evident up to the reproductive stage (70-day-old plants), where FC_14_, FC_17_, and FC_9_ continued to significantly enhance CCI values by 143.7, 134.7, and 128.6% over Ortho-P and by 231,  218.8, and 210.4% over RP uninoculated treatment, respectively (Supplementary Table S3). This remarkable continuity underscores the sustained positive influence of FC on chlorophyll content, marking a promising trajectory from the early vegetative phase to the reproductive stage of wheat plant development.

The impact of FC on wheat plant development was also consistent across growth stages, as evident in the shoot height (SH). In the vegetative stage, FC_14_, FC_17_, FC_9_, FC_8_, and FC_6_ exhibited high SH, accounting for 33.2, 27.5, 20, and 9.4% increases over the Ortho-P treatment and even more (70.7, 63.4, 53.7, 40.2, and 40.2%), respectively, when compared with the RP uninoculated treatment. Transitioning to the reproductive stage (70-day-old plants), FC_14_, FC_17_, FC_9_, and FC_8_ confirmed their positive effect on SH, with an average increase of 20.1, 10.4, 8.3, and 2.7 % over ortho-P and far higher (26.9, 16.6, 14.4, and 8.4%) as compared with RP uninoculated treatment, respectively (Supplementary Table S3).

#### Post-harvest parameters

Only a few FC increased the SDW of 30-day-old plants, notably FC_14_ and FC_17_ having the highest SDW values of 0.34 g and 0.33 g, respectively, representing significant increases of 12.2% and 11.1% compared with Ortho-P treatment (0.30 g), and far more (65.57% and 63.9% increase) when compared with RP uninoculated treatment (0.21 g) (Table 2). Similarly, FC positively influenced SDW of 70-day-old plants, especially FC_14_ (1.54 g), FC_17_ (1.53 g), FC_9_ (1.50 g), and FC_8_ (1.48 g), with an average increase of 30.9, 30.1, 27.9, and 25.8%, respectively, over Ortho-P and an important increase (52.0, 51.0, 48.5, and 46.0%, respectively) compared with RP uninoculated treatment (1.01 g) (Table 2). These results provide further evidence of the growth-promoting activity of FC, mainly FC_14_ and FC_17_.

**Table 2.** Dry weights of shoots and roots and root morphological traits of 30- and 70-day-old wheat plants inoculated with FC versus uninoculated treatments under RP supply

	30-day-old seedling					70-day old plants				
Treatments	SDW (g)	RDW (g)	RL (cm)	RSA (cm2)	RV (cm3)	SDW (g)	RDW (g)	RL (cm)	RSA (cm2)	RV (cm3)
Ortho-P	0.3 ab	0.3 c	239.0 ab	22.2 d–h	0.2 a–d	1.2 bc	0.5 d	372.0 abc	61.0 def	0.6 b–e
RP	0.2 d–g	0.2 de	144.6 fg	19.7 fgh	0.1 d–g	1.0 cde	0.4 ef	281.3 cd	59.9 def	0.5 e–j
P_0_	0.2 f–h	0.2 ghi	80.7 h	7.6 i	0.1 g	0.6 fh	0.3 hij	179.9 d	29.4 hi	0.3 jkl
FC_2_	0.2 a	0.2 de	140.3 cd	14.6 ijk	0.1 d–g	1.0 cde	0.4 e	350.2 abc	56.7 d–g	0.4 e–k
FC_3_	0.2 a	0.2 de	155.1 bc	18.0 e–j	0.1 d–g	1.1 c	0.4 e	332.2 cd	68.0 c–f	0.5 d–i
FC_4_	0.2 ab	0.2 fgh	108.4 d	12.0 jkl	0.1 g	0.7 d–h	0.3 hi	321.5 cd	52.3 d–g	0.3 h–l
FC_6_	0.3 abc	0.3 c	170.6 b	24.0 a–e	0.2 b–e	1.2 bc	0.5 d	389.0 abc	49.5 efg	0.4 f–l
FC_8_	0.3 bcd	0.3 b	180.5 b	24.9 bcd	0.2 a–e	1.5 ab	0.6 c	315.0 cd	30.1 hi	0.2 f–l
FC_9_	0.4 b–e	0.4 a	190.7 b	25.7 abc	0.2 a–e	1.5 a	0.6 b	314.8 cd	52.0 d–g	0.4 f–k
FC_13_	0.2 b–e	0.2 f	167.9 b	20.8 b–i	0.2 cde	1.1 bc	0.4 ef	323.3 cd	52.5 d–g	0.4 f–k
FC_14_	0.4 b–f	0.4 a	250.8 a	28.3 a	0.2 a	1.5 a	0.7 a	515.1 a	97.7 a	0.8 ab
FC_15_	0.2 b–f	0.2 f	153.7 bc	16.5 g–j	0.1 d–g	1.0 c–g	0.3 gh	363.3 abc	68.0 c–f	0.5 d–i
FC_17_	0.4 b-f	0.4 a	240.8 a	26.8 ab	0.2 abc	1.5 a	0.7 ab	511.1 ab	100.7 a	0.9 a
FC_18_	0.2 b–g	0.2 fgh	158.2 bc	15.8 hij	0.1 d–g	0.6 gh	0.3 hi	263.7 cd	23.4 i	0.2 l
FC_21_	0.2 c–g	0.2 d	77.2 e	17.4 f–j	0.2 cde	1.0 cde	0.4 fg	375.6 abc	50.6 d–g	0.3 h–l
FC_22_	0.2 c–g	0.2 fgh	77.2 e	8.9 kl	0.1 g	1.0 c–f	0.4 ef	395.1 abc	61.0 def	0.5 e–j
FC_26_	0.2 c–g	0.2 e	168.9 b	18.8 d–i	0.1 def	1.1 c	0.4 ef	373.9 abc	72.1 bcd	0.7 a–d
FC_28_	0.2 d–h	0.2 f–i	148.0 bc	16.0 hij	0.1 efg	0.9 c–g	0.3 ijk	392.2 abc	61.3 def	0.5 d–i
FC_R1_	0.2 d–h	0.1 hi	143.2 bc	21.5 b–h	0.1 cde	0.9 c–g	0.3 k	335.7 cd	89.6 ab	0.8 abc
FC_R2_	0.1 d–h	0.1 j	167.9 b	19.6 c–i	0.1 c–f	0.7 e–h	0.2 l	318.9 cd	47.6 gfh	0.4 g–l
FC_R3_	0.1 d–h	0.1 j	166.0 b	20.1 c–i	0.1 d–g	0.4 h	0.2 l	364.6 abc	53.8 d–g	0.4 e–k
FC_R4_	0.1 e–h	0.1 ij	168.9 ab	15.6 hij	0.1 d–g	0.5 h	0.3 jk	316.2 cd	37.9 ghi	0.3 d–h
FC_R5_	0.2 fh	0.2 f	176.2 b	18.5 d–i	0.1 d–g	1.0 c–f	0.4 ef	366.3 abc	67.8 c–f	0.5 d–h
FC_R6_	0.2 gh	0.2 fgh	160.8 bc	22.7 a–g	0.1 cde	1.1 c	0.4 ef	341.5 cd	62.3 def	0.6 c–f
FC_R7_	0.2 gh	0.2 fg	168.8 b	17.2 f–g	0.1 d–g	1.2 bc	0.4 ef	355.5 abc	70.7 b–e	0.6 c–g
FC_G_	0.2 h	0.2 f	155.1 bc	19.8 c–i	0.1 cde	1.1 cd	0.4 efg	345.8 bcd	85.9 abc	0.4 f–k

SDW, shoot dry weight; RDW, root dry weight; RL, root length; RSA, root surface area; RV, root volume; Ortho-P, fertilized with orthophosphates; RP, fertilized with rock phosphate; P_0_, unfertilized and uninoculated; FC, niche-conserved FC; FC_R1_–FC_R7_, intra-region FC; FC_G_, global (inter-region) FC.

Values are means ±SD. Different letters indicate values that are statistically different at *P*<0.05.

FC increased RDW of 30-day-old plants including FC_14_, FC_9_, FC_17_, and FC_8_ with 22.9, 18.7, 16.7, and 9.4%, respectively, over Ortho-P and even more (81.5, 75.4, 72.3, and 61.5%, respectively) as compared with RP uninoculated treatment. Similarly, FC_14_, FC_17_, FC_9_, and FC_8_ increased RDW with 40.5, 34.9, 30.77, and 21.0%, respectively, over Ortho-P (0.49 g) and with 72.3, 65.4, 60.4, and 48.4% as compared with RP uninoculated treatment (Table 2).

In addition, the range of leaf area values for the FC treatments varied from 9.52 cm^2^ to 35.43 cm^2^, with the highest leaf area value among the inoculated 30-day-old plants being associated with FC_14_, with an increase of ~14.0% over Ortho-P and 65.5% compared with RP uninoculated treatment (Supplementary Table S3).

#### Morphological root parameters (RL, RSA, and RV)

FC inoculation significantly enhanced morphological root parameters (RL, RSA, and RV) of 30-day-old plants. FC_14_ and FC_17_ induced the highest RL (250.81 cm and 240.8 cm, respectively), representing increases of 5.3% and 1.1% as compared with Ortho-P, and 73.40% and 66.5% as compared with RP uninoculated treatments, respectively (Table 2). Secondly, FC_14_, FC_17_, FC_9_, FC_8_, FC_6_, and FC_R6_ significantly increased RSA by 27.6, 21.0, 15.8, 12.4, 8.5, and 2.4% compared with Ortho-P treatment and by 48.3, 40.7, 34.7, 30.7, 26.1, and 19.2% when compared with RP uninoculated treatment, respectively (Table 2). For RV, the highest value was attributed to FC_14_ with 0.24 cm^3^, an increase of 4.3% and 60.0% as compared with Ortho-P and RP uninoculated treatments, respectively (Table 2).

The positive influence of the FC on root morphological parameters extended to the reproductive stage (70-day-old plants), validating their significant effect on RL, RSA, and RV at different growth stages. Specifically, FC_14_, FC_17_, FC_22_, and FC_8_ significantly increased RL by 38.5, 37.4, 6.2, and 5.4%, compared with Ortho-P, and by 83.1, 81.7, 40.5, and 39.4% compared with RP uninoculated treatment, respectively (Table 2). Furthermore, FC_17_, FC_14_, FC_R1_, and FC_G_ positively impacted RSA, resulting in increases of 65.1, 60.1, 46.7, and 40.8% over Ortho-P and 68.1, 63.1, 49.6, and 43.4%, over RP uninoculated treatment, respectively (Table 2). Moreover, FC positively influenced RV, with the highest values recorded for FC_17_, FC_14_, FC_R1_, and FC_26_ increasing RV by 33.8, 24.6, 18.5, and 13.8% over Ortho-P and by 77.5, 65.3, 57.1, and 51.0% over RP uninoculated treatment, respectively (Table 2). These findings collectively highlight the comprehensive and positive impact of FC on the root morphology and growth of both 30- and 70-day-old plants. These findings not only confirm but strongly underscore the remarkable effectiveness of the FC, especially FC_14_ and FC_17_ as plant growth-promoting consortia. Plant growth, rooting parameters, and soil-available P content were significantly improved by FC_14_ and FC_17_ at both vegetative and reproductive stages compared with Ortho-P treatment. These improvements were even greater compared with the RP-amended treatment.

Notably, these improvements were particularly remarkable when contrasted with the P_0_ (uninoculated and non-amended control) which recorded the lowest values across all the parameters studied. Furthermore, some FC including FC_14_, FC_17_, FC_9_, FC_8_, and FC_6_ had the highest values in terms of all the growth and rooting parameters, as well as rhizosphere-available P of 30-day-old wheat plants. It also should be highlighted that the performance of FC_14_ and FC_17_ remained highest for all the parameters studied at both the vegetative and reproductive stages.

#### Rhizosphere-available P content

**Fig 7.**
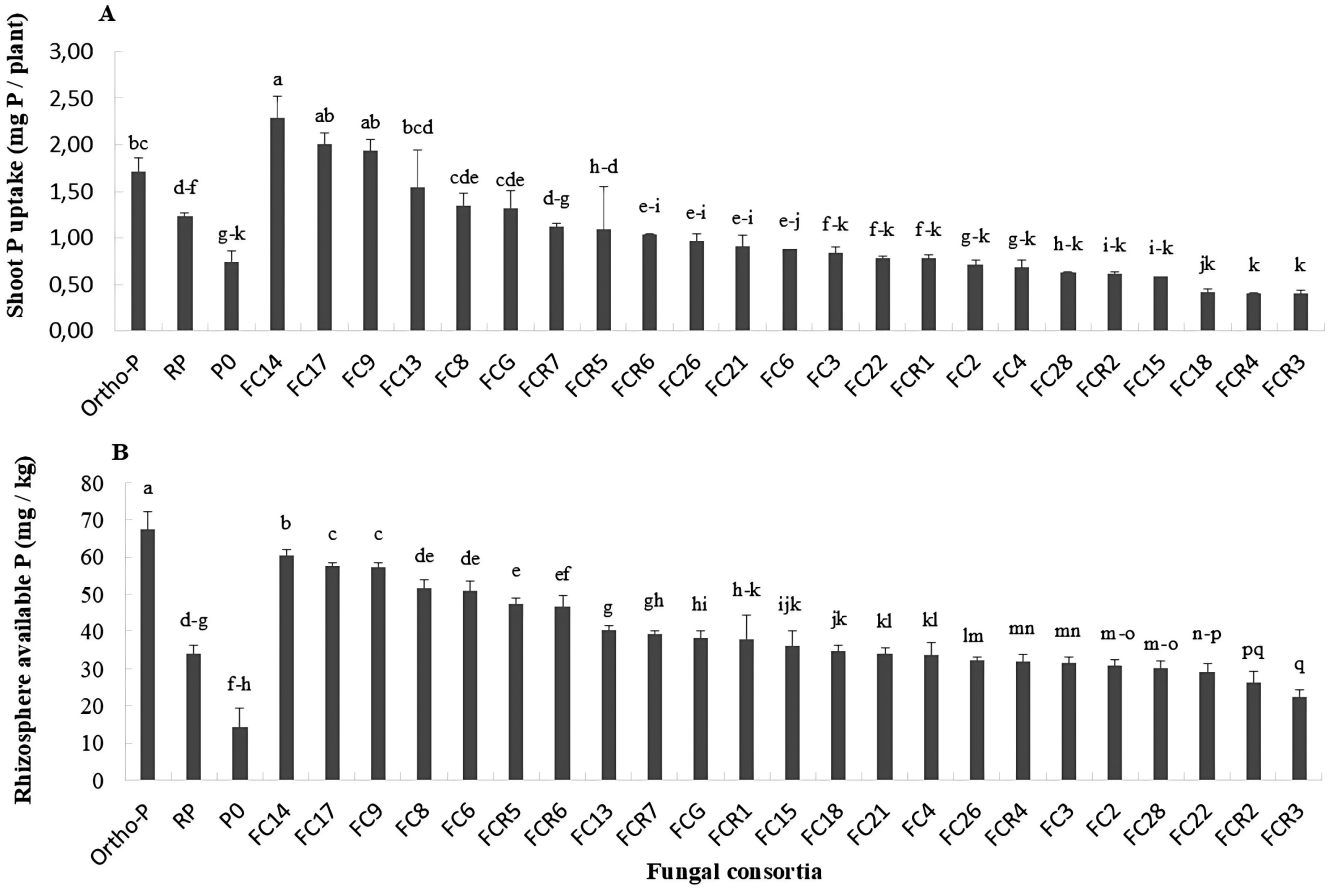
Shoot P uptake (A) and rhizosphere-available P (B) of 75- and 30-day-old wheat plants, inoculated with FC versus uninoculated treatments (Ortho-P, RP, and P_0_) under RP supply. FC, zone FC; FC_R1_–FC_R7_, intra-region FC; FC_G_, global and inter-region FC; Ortho-P, fertilized with orthophosphates; RP, fertilized with rock phosphate; and P_0_, unfertilized and uninoculated. Bars are displayed in descending order from left to right, except those of uninoculated treatments on the left. The results are means ±SD (*n*=4). Different letters indicate values that are statistically different at *P*<0.

In the 30-day-old rhizosphere soil, the uninoculated treatments (Ortho-P, RP, and P_0_) had relatively stable P concentrations, while FC treatments ranged between 22.5 mg kg^–1^ and 60.4 mg kg^–1^. Notably, FC_14_, FC_17_, FC_9_, and FC_8_ treatments had the highest P concentrations, with 60.4, 57.5, 57.4, 51.4, and 50.8 mg kg^–1^, respectively (Fig. 7B). This trend continued in 70-day-old rhizosphere soil, where FC, particularly FC_17_, FC_8_, FC_9_, and FC_14_, positively influenced soil-available P content (55.4, 54.3, 53.8, and 5.00 mg kg^–1^, respectively) over uninoculated treatments (Supplementary Table S3). For the majority of FC, the concentration of 70-day-old rhizosphere-available P was lower than in the rhizosphere of 30-day-old plants, which indicated a decrease in available P concentrations associated with increased plant growth.

#### Shoot P, K, and N uptake of 70-day-old wheat plants

FC positively influenced the shoot P uptake of 70-day-old plants, especially FC_14_, FC_17_, and FC_9_, resulting in increases of 33.4, 16.7, and 12.7% over Ortho-P and 85.7, 62.4, and 56.9% over RP uninoculated treatment, respectively (Fig. 7A). In addition, FC positively influenced the shoot K uptake of 70-day-old plants, especially FC_9_, FC_14_, FC_17_, and FC_8_, resulting in increases of 57.54, 40.98, 40.55, and 38.44% over Ortho-P and 108.2, 86.3, 85.7, and 83.0% over RP uninoculated treatment, respectively (Supplementary Table S3). Moreover, FC also enhanced shoot N uptake of 70-day-old plants, notably FC_17_, FC_14_, FC_8_, and FC_9_ displaying an increase of 61.3, 58.1, 57.0, and 34.4% over Ortho-P and 54.0, 51.0, 49.9, and 28.3% as compared with RP uninoculated treatment, respectively (Supplementary Table S3). These findings underscore the efficacy of FC in enhancing shoot P, K, and N uptake, surpassing the performance of traditional phosphate sources and RP.

## Discussion

The present study combined both culture-dependent and culture-independent methods to bioprospect wheat rhizoplane-associated fungi at both diversity and functional levels across seven agricultural regions in Morocco. Detailed insights into total community and function of rhizoplane fungal species were deciphered and successfully put together into a consortium construction-oriented isolation approach that simulates the intricate natural fungal communities (niche-conserved). This study explored the diversity of wheat rhizoplane fungal communities and demonstrated its functional potential. The large-scale bioprospection adopting the niche-conserved approach allowed for isolation of 20 distinct fungal species from 28 different niches (zones) within seven regions (Fig. 3). The low count of isolated fungi (20) from the rhizoplane indicates lower fungal diversity in this compartment compared with the rhizosphere or bulk soil. This aligns with findings by Park *et al*. (2021), who noted fewer fungal taxa in rhizoplane samples, suggesting selective pressures from the host plant. This consistency matches previous research illustrating a lowering diversity gradient from bulk soil to the rhizoplane (Barton and Northup, 2011). Despite its reduced fungal diversity, the focus on the rhizoplane is supported by the increased PGP activities rather than diversity. Moreover, ITS amplicon sequencing across the seven agricultural regions resulted in the identification of 4456 ASVs (Fig. 4), which appears to be lower compared with the rhizosphere and bulk soil in other studies (Lang *et al.*, 2019), which could be due to various factors, including selective pressures from the host plant, differences in soil types, or geographic location (Lang *et al.*, 2019; Pantigoso *et al.*, 2022). This highlights the need for niche-specific research (rhizoplane here) and holistic synthetic consortia construction approaches to fully explore the microbial diversity and functions within different niches around the roots and their contributions to ecosystem processes.

The niche-conserved approach adopted an FC-oriented methodology that generated 28 niche-conserved FC, as well as seven intra-region and one inter-region constructed FC. Refining FC construction by retaining only non-pathogenic and compatible isolates generated 23 FC, comprising 15 niche-conserved (or zone FC), seven intra-region, and one inter-region constructed FC (Supplementary Table S1). FC application on wheat plants demonstrated the effectiveness of these FC, in particular two niche-conserved constructed FC (FC_14_ and FC_17_) which include F_9_, F_11_, and F_12_ fungal isolates, in improving wheat growth at vegetative and reproductive stages, as well as enhancing P availability. Integrating the removal of pathogenic and non-biocompatible species from FC construction was done to ensure safety and positive synergy within constructed consortia (Benbrik *et al.*, 2020). *Penicillium cyclopium* strain CECT 2264 ‘F_8_’, *F. graminearum* isolate Fs-LA1 ‘F_10_’, and *A. infectoria* strain ‘F_19_’ were eliminated as they proved to be pathogenic. According to Leplat *et al.* (2012), *F.* graminearum isolate Fs-LA1 was reported as wheat head blight agent. *Alternaria infectoria* species have been reported as a phytopathogen as well as an opportunistic human pathogen (Knutsen *et al.*, 2012; Silva *et al.*, 2014). The strategy, integrating safety measures and biocompatibility verification, contributes to the development of FC that are not only diverse and efficient but also synergistic and safe.

The testing of FC capacity on 30- and 70-day-old plants showed improvement in plant growth, root morphology, and shoot nutrient (P, K, and N) uptake parameters (Supplementary Table S3). FC_14_ and FC_17_ were the best performing FC across all studied parameters at both the vegetative and reproductive growth stages. The effectiveness of FC_14_ and FC_17_ can be attributed to the presence of specific fungal species within their composition, namely *T. verruculosus* strain n (F_9_), *Talaromyces* sp. clone SF_430 (F_11_), and *R. arrhizus* CMRC 585 (F_12_). These fungal species have demonstrated exceptional performance in enhancing both above-ground development and root morphology in 20-day-old wheat seedlings. Notably, they exhibit significant *in vitro* PGP properties including PS, OA production, phosphatase production, KS, and ZS, contributing to the overall efficiency of the FC they compose (Table 1). The functional characterization of the fungal isolates, notably F_11_ and F_12_, revealed significant potential for PS, with a significant correlation to pH lowering (Supplementary Table S4). This capability aligns with established knowledge, emphasizing the role of pH lowering and acidification in P solubilization (Bargaz *et al.*, 2021). This highlights their potential as beneficial agents in promoting plant growth and enhancing nutrient availability in soil, which has implications for agricultural and ecological applications (Cheng *et al.*, 2022). Moreover, previous research indicates that species from the genus *Talaromyces* are well documented for their PGP traits, including P solubilization, nitrogen fixation, and hormone production, supporting their efficacy in agricultural and ecological applications (Cerullo *et al.*, 2018; Sembiring *et al.*, 2018; Goh *et al.*, 2020; El-Shahir *et al.*, 2021). *Talaromyces* sp. clone SF_430 was also reported for its antifungal activity and heavy metal absorption (Wakelin *et al.*, 2007; Radhakrishnan *et al.*, 2014). Additionally, the presence of F_9_, F_11_, and F_12_ in different niches suggests that they possess a degree of resilience and adaptability in terms of ecological niches, making them a successful and widespread species (Barton and Northup, 2011). Looking at the diversity of constructed FC, niche-conserved FC consist of just two or three fungal species, whereas intra- and inter-region FC contain multiple species. Their *in planta* assessments showed that niche-conserved FC, mainly FC_14_ and FC_17_, performed better. This indicates that even with fewer species, niche-conserved FC exhibit significant effectiveness, emphasizing the importance of targeted fungal species with specific PGP functions rather than sheer diversity.

Additionally, FC_14_, FC_17_, FC_9_, and FC_8_ treatments resulted in a significant increase in soil-available P concentrations, which might be linked to improved growth of 30- and 70-day-old plants under RP supply that was made accessible for plant uptake. This also demonstrated a clear connection between the enhanced growth parameters observed in wheat seedlings in response to inoculation with FC_14_ and FC_17_, owing to their ability to release available P from RP in the rhizosphere soil as compared with uninoculated RP treatment. Furthermore, enhanced shoot P uptake within FC-inoculated plants confirms this conclusion particularly for potent FC (FC_14_ and FC_17_).

In most studies, the emphasis is on monoculture microbial methods, which contrasts with field settings where the presence of the local microbiome naturally results in significant competition. This competition is believed to be the reason behind the failure of single-strain inoculations in real-field conditions (Grosskopf and Soyer, 2014). Instead, designing synthetic microbial communities may greatly increase the resilience of microorganisms especially in an open system (Schmitz *et al.*, 2022). Nonetheless, there remains uncertainty about an effective method for constructing functional microbial consortia. Here we propose a niche-conserved approach that employed a consortium-oriented isolation strategy. This strategy (multi-PGP traits) involved three culture media, PS and NF (critical for plant health), as well as other PGP traits that are the desired ecological functions in our targeted synthetic community (FC). Through this strategy, we aimed to preserve the intra-niche diversity and functions. The choice of PS, NF, and PGP functions aligns with their fundamental roles in nutrient uptake, plant metabolism, and plant growth promotion (Shcherbak *et al.*, 2014; Guo *et al.*, 2022). This multi-PGP trait strategy ensures capturing intra-niche community with holistic functional diversity and high survival ability, which is often overlooked when using traditional isolation with single-culture medium methods or when focusing on a single strain (Schmitz *et al.*, 2022). The design of synthetic microbial consortia, mimicking natural ecosystems, tailors communities with specific functions, promising advancements in biotechnology. Specifically, it enables the production of effective crop inoculants for boosting yield and meeting global food demand (Grosskopf and Soyer, 2014; Sharma *et al.*, 2023).

Moreover, the distribution of isolated fungi across regions exhibited an interesting pattern, notably with Sidi Kacem displaying both the highest total and functional diversity, demonstrating substantial functional fungal richness and suggesting a complex and dynamic ecosystem. Conversely, Azilal, despite higher total diversity, had lower beneficial functional diversity, raising questions about species cultivability, ecological roles, and interactions in that region. Additionally, regional distribution underscores the unique functional fungal communities that can be found in specific geographic areas, shedding light on the ecological significance of fungi in these regions and offering potential insights into their roles in various ecosystems. This multi-disciplinary strategy, covering culture- and non-culture-based methods, explores species richness and delves into ecological significance and niche performance. Furthermore, the success in capturing different species indigenous to a specific geographic area underscores the value of the methodology in regional biodiversity studies and ecological function within the niche level. Comparing the taxonomic identification of ITS region ASVs with fungal isolate sequences with the UNITE database classification offers unique insights into the current state of fungal classification. In general, classifications showed consistency, with genera being the same for ASVs classified by both databases. There were some discrepancies with the genus *Fusarium*, whereby nine out of 42 ASVs were unidentified to phylum level by UNITE. Additionally, the genus *Neodidymelliopsis* was not detected in ASVs classified by UNITE but was found to be abundant when classified by fungal ITS isolate sequences. The *Didymellaceae* family is one of the most species-rich families in the fungal kingdom (Chen *et al.*, 2015, 2017), with ~50 genera reported on NCBI taxonomy, 27 of which are represented by sequences in the UNITE database (Nilsson *et al.*, 2018); *Neodidymelliopsis*, however, is only represented by one sequence in the UNITE database which could explain why this genus was not detected in our DNA samples. *Didymella* and *Neoascochyta* were the only two genera of the *Didymellaceae* family detected by UNITE, represented by 55 and 15 ASVs, respectively. To the best of our knowledge, this is the first study to directly link cultured fungal isolate ITS sequences with culture-independent amplicon data, with previous studies using both methods in parallel (Selbmann *et al.*, 2021; Zheng *et al.*, 2021).

In conclusion, this study used multi-disciplinary research (microbiology, and genomic and plant ecophysiology) to validate a novel FC construction methodology based on the niche-conserved approach, hence marking a unique contribution by being the first to explore the fungal diversity of the wheat rhizoplane, functions, as well as niche inter-species interactions, and their use in a niche-conserved consortium construction approach for P acquisition and plant growth promotion, leading to the development of a efficient synthetic fungal community. Despite their lower diversity, niche-conserved constructed FC (mainly FC_14_ and FC_17_) demonstrated superior performance compared with intra- and inter-region FC in promoting plant growth and enhancing P acquisition efficiency. Their consistent effectiveness across various growth parameters and stages underlines their significant impact on wheat growth promotion. Such a novel focus on rhizoplane fungi as part of this multi-disciplinary study enhanced our understanding of microbial interactions around plant roots. Thus, the success of a niche-conserved approach in designing PGP-effective FC presents promising prospects in synthetic biology, underlining its potential for further advances in this field. For future research orientations, further validation of the niche-conserved approach and evaluation is recommended. This involves conducting additional studies using this approach to confirm its efficacy by testing microbial synthetic communities potentially beneficial for different crops and varieties under different soil and climate conditions. Such research efforts would contribute significantly to the advancement of synthetic microbial solutions aimed at sustaining plant productivity.

## Supplementary data

The following supplementary data are available at *JXB* online.

Fig. S1. Rarefaction curves showing the number of fungal ASVs observed against the number of sequences.

Table S1. Fungal composition of zone (niche conservatism), intra-region FC, and the inter-region (global) FC.

Table S2. ASVs classified to genus level.

Table S3. Above-ground plant parameters, rhizosphere-available P, and shoot K and N uptake of 30- and 70-day-old wheat plants inoculated with FC versus uninoculated treatments under RP supply.

Table S4. Pearson correlation between soluble P, pH, and gluconic acid concentration of fungal isolates (F_1_–F_20_) grown in NBRIP liquid medium supplemented with TCP.

eraf042_suppl_Supplementary_Figure_S1_Tables_S1-S4

## Data Availability

All Illumina sequence data from this study are available in the National Center for Biotechnology Information (NCBI) under accession number PRJNA1067980. Data for the amplicon sequence analysis are deposited under the CC-BY 4.0 license in a Rothamsted Repository (DOI https://doi.org/10.23637/dgzm59o5). Scripts for the analysis in QIIME2 and RStudio are freely available under the Apache 2-0 license and have been deposited in a Zenodo repository (https://doi.org/10.5281/zenodo.14204978).

## References

[CIT0001] Aallam Y , Dhiba D, El Rasafi T, Lemriss S, Haddioui A, Tarkka M, Hamdali H. 2022. Growth promotion and protection against root rot of sugar beet (*Beta vulgaris* L.) by two rock phosphate and potassium solubilizing *Streptomyces* spp. under greenhouse conditions. Plant and Soil 472, 407–420.

[CIT0002] Anwar H , Wang X, Hussain A, Rafay M, Ahmad M, Latif M, Jamshaid MU, Khalid I, Dar A, Mustafa A. 2021. Comparative effects of bio-wastes in combination with plant growth-promoting bacteria on growth and productivity of okra. Agronomy 11, 2065.

[CIT0003] Bargaz A , Elhaissoufi W, Khourchi S, Benmrid B, Borden KA, Rchiad Z. 2021. Benefits of phosphate solubilizing bacteria on belowground crop performance for improved crop acquisition of phosphorus. Microbiological Research 252, 126842. 34438221 10.1016/j.micres.2021.126842

[CIT0004] Bargaz A , Lyamlouli K, Chtouki M, Zeroual Y, Dhiba D. 2018. Soil microbial resources for improving fertilizers efficiency in an integrated plant nutrient management system. Frontiers in Microbiology 9, 1–25. 30108553 10.3389/fmicb.2018.01606 PMC6079243

[CIT0006] Baris O , Sahin F, Turan M, Orhan F, Gulluce M. 2014. Use of plant-growth-promoting rhizobacteria (PGPR) seed inoculation as alternative fertilizer inputs in wheat and barley production. Communications in Soil Science and Plant Analysis 45, 2457–2467.

[CIT0007] Barton LL , Northup DE. 2011. Microbial ecology. Oxford: Wiley-Blackwell, John Wiley & Sons.

[CIT0008] Benbrik B , Elabed A, El Modafar C, Douira A, Amir S, Filali-Maltouf A, El Abed S, El Gachtouli N, Mohammed I, Koraichi SI. 2020. Reusing phosphate sludge enriched by phosphate solubilizing bacteria as biofertilizer: growth promotion of *Zea mays*. Biocatalysis and Agricultural Biotechnology 30, 101825.

[CIT0009] Benmrid B , Ghoulam C, Zeroual Y, Kouissni L, Bargaz A. 2023. Bioinoculants as a means of increasing crop tolerance to drought and phosphorus deficiency in legume–cereal intercropping systems. Communications Biology 6, 1016. 37803170 10.1038/s42003-023-05399-5 PMC10558546

[CIT0010] Bokulich NA , Kaehler BD, Rideout JR, Dillon M, Bolyen E, Knight R, Huttley GA, Gregory Caporaso J. 2018. Optimizing taxonomic classification of marker-gene amplicon sequences with QIIME 2’s q2-feature-classifier plugin. Microbiome 6, 90. 29773078 10.1186/s40168-018-0470-z PMC5956843

[CIT0011] Bolyen E , Rideout JR, Dillon MR, et al 2019. Reproducible, interactive, scalable and extensible microbiome data science using QIIME 2. Nature Biotechnology 37, 852–857. 10.1038/s41587-019-0209-9 PMC7015180 31341288

[CIT0012] Callahan BJ , McMurdie PJ, Rosen MJ, Han AW, Johnson AJ, Holmes SP. 2016. DADA2: high-resolution sample inference from Illumina amplicon data. Nature Methods 13, 581–583. 27214047 10.1038/nmeth.3869 PMC4927377

[CIT0018] Cerullo G , Houbraken J, Granchi Z, Pepe O, Varriale S, Ventorino V, Chin-A-Woeng T, Meijer M, de Vries RP, Faraco V. 2018. Draft genome sequence of *Talaromyces adpressus*. Genome Announcements 6, e01430-17. 29326215 10.1128/genomeA.01430-17 PMC5764939

[CIT0013] Chandio AA , Gokmenoglu KK, Ahmad M, Jiang Y. 2022. Towards sustainable rice production in Asia: the role of climatic factors. Earth Systems and Environment 6, 1–14.

[CIT0015] Chen Q , Hou LW, Duan WJ, Crous PW, Cai L. 2017. Didymellaceae revisited. Studies in Mycology 87, 105–159. 28706324 10.1016/j.simyco.2017.06.002 PMC5498420

[CIT0016] Chen Q , Jiang JR, Zhang GZ, Cai L, Crous PW. 2015. Resolving the Phoma enigma. Studies in Mycology 82, 137–217. 26955202 10.1016/j.simyco.2015.10.003 PMC4774273

[CIT0014] Chen S , Zhou Y, Chen Y, Gu J. 2018. fastp: an ultra-fast all-in-one FASTQ preprocessor. Bioinformatics 34, i884–i890. 30423086 10.1093/bioinformatics/bty560 PMC6129281

[CIT0017] Cheng S , Jiang JW, Tan LT, Deng JX, Liang PY, Su H, Sun Z-X, Zhou Y. 2022. Plant growth-promoting ability of mycorrhizal fusarium strain KB-3 enhanced by its IAA producing endohyphal bacterium, *Klebsiella aerogenes*. Frontiers in Microbiology 13, 2X. 10.3389/fmicb.2022.855399 PMC9051524 35495715

[CIT0019] Crossay T , Majorel C, Redecker D, Gensous S, Medevielle V, Durrieu G, Cavaloc Y, Amir H. 2019. Is a mixture of arbuscular mycorrhizal fungi better for plant growth than single-species inoculants? Mycorrhiza 29, 325–339. 31203456 10.1007/s00572-019-00898-y

[CIT0020] de Mendiburu F. 2021. agricolae: statistical procedures for agricultural research. R package version 1.3-5. https://CRAN.R-project.org/package=agricolae

[CIT0021] Edgar RC , Haas BJ, Clemente JC, Quince C, Knight R. 2011. UCHIME improves sensitivity and speed of chimera detection. Bioinformatics 27, 2194–2200. 21700674 10.1093/bioinformatics/btr381 PMC3150044

[CIT0022] Elhaissoufi W , Khourchi S, Saidi R, Ibnyasser A, Haddine M, Ghani R, Zeroual Y, Rchiad Z, Ghoulam C, Bargaz A. 2024. Inoculation with phosphate solubilizing bacterial consortia enhanced rock P agronomic efficiency and yield of wheat under low P conditions. Journal of Plant Growth Regulation. https://doi.org/10.1007/s00344-024-11350-7

[CIT0023] El-Shahir AA , El-Tayeh NA, Ali OM, Abdel-Latef AA, Loutfy N. 2021. The effect of endophytic *Talaromyces pinophilus* on growth, absorption and accumulation of heavy metals of *Triticum aestivum* grown on sandy soil amended by sewage sludge. Plants 10, 2659. 34961130 10.3390/plants10122659 PMC8704920

[CIT0024] Faith DP. 1992. Conservation evaluation and phylogenetic diversity. Biological Conservation 61, 1–10.

[CIT0025] Felsenstein J. 1985. Confidence limits on phylogenies: an approach using the bootstrap. Evolution 39, 783–791. 28561359 10.1111/j.1558-5646.1985.tb00420.x

[CIT0026] Goh YK , Marzuki NF, Tuan Pa TNF, Goh TK, Kee ZS, Goh YK, et al 2020. Biocontrol and plant-growth-promoting traits of *Talaromyces apiculatus* and *Clonostachys rosea* consortium against ganoderma basal stem rot disease of oil palm. Microorganisms 8, 1138. 32731441 10.3390/microorganisms8081138 PMC7463586

[CIT0027] Grosskopf T , Soyer OS. 2014. Synthetic microbial communities. Current Opinion in Microbiology 18, 72–77. 24632350 10.1016/j.mib.2014.02.002 PMC4005913

[CIT0028] Gujar PD , Bhavsar KP, Khire JM. 2013. Effect of phytase from *Aspergillus niger* on plant growth and mineral assimilation in wheat (*Triticum aestivum* Linn.) and its potential for use as a soil amendment. Journal of the Science of Food and Agriculture 93, 2242–2247. 23355258 10.1002/jsfa.6032

[CIT0029] Guo C , Liu X, He X. 2022. A global meta-analysis of crop yield and agricultural greenhouse gas emissions under nitrogen fertilizer application. The Science of the Total Environment 831, 154982. 35381236 10.1016/j.scitotenv.2022.154982

[CIT0030] Harman GE , Petzoldt R, Comis A, Chen J. 2004. Interactions between *Trichoderma harzianum* strain T22 and maize inbred line Mo17 and effects of this interaction on diseases caused by *Pythium ultimum* and *Colletotrichum graminicola*. Phytopathology 94, 147–153. 18943537 10.1094/PHYTO.2004.94.2.147

[CIT0031] Hett J , Neuhoff D, Döring TF, Masoero G, Ercole E, Bevivino A. 2022. Effects of multi-species microbial inoculants on early wheat growth and litterbag microbial activity. Agronomy 12, 899.

[CIT0032] Hossain MM , Sultana F, Islam S. 2017. Plant growth-promoting fungi (PGPF): phytostimulation and induced systemic resistance. In: Singh D, Singh H, Prabha R, eds. Plant–microbe interactions in agro-ecological perspectives. Singapore: Springer, 978–981.

[CIT0033] Hyakumachi M. 1994. Plant-growth-promoting fungi from turf grass rhizosphere with potential for disease suppression. Soil Microorganisms 44, 53–68.

[CIT0034] Kamath A , Shukla A, Saiyed T, Bhatt S, Rathod H, Makwana V, Soni D, Banerjee S, Patel D. 2023. Bioinoculants: the agrarian avengers. Symbiosis 91, 151–166.

[CIT0035] Katoh K , Misawa K, Kuma K, Miyata T. 2002. MAFFT: a novel method for rapid multiple sequence alignment based on fast Fourier transform. Nucleic Acids Research 30, 3059–3066. 12136088 10.1093/nar/gkf436 PMC135756

[CIT0036] Kembel SW , Cowan PD, Helmus MR, Cornwell WK, Morlon H, Ackerly DD, Blomberg SP, Webb CO. 2010. Picante: R tools for integrating phylogenies and ecology. Bioinformatics 26, 1463–1464. 20395285 10.1093/bioinformatics/btq166

[CIT0037] Khourchi S , Elhaissoufi W, Loum M, et al 2022. Phosphate solubilizing bacteria can significantly contribute to enhance P availability from polyphosphates and their use efficiency in wheat. Microbiological Research 262, 127094. 35749891 10.1016/j.micres.2022.127094

[CIT0038] Kivlin SN , Hawkes CV, Papeş M, Treseder KK, Averill C. 2021. The future of microbial ecological niche theory and modeling. New Phytologist 231, 508–511. 34132414 10.1111/nph.17373

[CIT0039] Klaic R , Plotegher F, Ribeiro C, Zangirolami TC, Farinas CS. 2017. A novel combined mechanical–biological approach to improve rock phosphate solubilization. International Journal of Mineral Processing 161, 50–58.

[CIT0040] Knutsen AP , Bush RK, Demain JG, et al 2012. Fungi and allergic lower respiratory tract diseases. Journal of Allergy and Clinical Immunology 129, 280–291. 22284927 10.1016/j.jaci.2011.12.970

[CIT0041] Lahti L , Shetty S. 2012–2019. R package version. 1.19.1. https://github.com/microbiome/microbiome

[CIT0042] Lang M , Bei S, Li X, Kuyper TW, Zhang J. 2019. Rhizoplane bacteria and plant species co-determine phosphorus-mediated microbial legacy effect. Frontiers in Microbiology 10, 2856. 31921037 10.3389/fmicb.2019.02856 PMC6914688

[CIT0044] Leplat J , Friberg H, Abid M, Steinberg C. 2012. Survival of *Fusarium graminearum*, the causal agent of Fusarium head blight. A review. Agronomy for Sustainable Development 33, 97–111.

[CIT0045] Lindemann SR , Bernstein HC, Song HS, Fredrickson JK, Fields MW, Shou W, Johnson DR, Beliaev AS. 2016. Engineering microbial consortia for controllable outputs. The ISME Journal 10, 2077–2084. 26967105 10.1038/ismej.2016.26 PMC4989317

[CIT0046] Love MI , Huber W, Anders S. 2014. Moderated estimation of fold change and dispersion for RNA-seq data with DESeq2. Genome Biology 15, 550. 25516281 10.1186/s13059-014-0550-8 PMC4302049

[CIT0047] Magoč T , Salzberg SL. 2011. FLASH: fast length adjustment of short reads to improve genome assemblies. Bioinformatics 27, 2957–2963. 21903629 10.1093/bioinformatics/btr507 PMC3198573

[CIT0048] Martin M. 2011. Cutadapt removes adapter sequences from high throughput sequencing reads. EMBnet Journal 17, 10–12.

[CIT0049] Matsuoka S , Sugiyama Y, Nagano M, Doi H. 2022. Influence of DNA extraction kits on freshwater fungal DNA metabarcoding. PeerJ 10, e13477. 35651749 10.7717/peerj.13477 PMC9150701

[CIT0050] McMurdie PJ , Holmes S. 2013. phyloseq: an R package for reproducible interactive analysis and graphics of microbiome census data. PLoS One 8, e61217. 23630581 10.1371/journal.pone.0061217 PMC3632530

[CIT0051] Mikryukov V. 2022. metagMisc: miscellaneous functions for metagenomic analysis. R package version 0.0.4.[Computer software] https://github.com/vmikk/metagMisc.

[CIT0052] Murphy J , Riley JP. 1962. A modified single solution method for the determination of phosphate in natural waters. Analytica Chimica Acta 27, 31–36.

[CIT0053] Nawaz A , Shahbaz M, Asadullah, Imran A, Marghoob MU, Imtiaz M, Mubeen F. 2020. Potential of salt tolerant PGPR in growth and yield augmentation of wheat (*Triticum aestivum* L.) under saline conditions. Frontiers in Microbiology 11, 2019. 33117299 10.3389/fmicb.2020.02019 PMC7562815

[CIT0054] Nei M , Kumar S. 2000. Molecular evolution and phylogenetics. Oxford: Oxford University Press.

[CIT0055] Nilsson RH , Larsson KH, Taylor AFS, et al 2019. The UNITE database for molecular identification of fungi:handling dark taxa and parallel taxonomic classifications. Nucleic Acids Research 47, D259–D264. 10.1093/nar/gky1022 PMC6324048 30371820

[CIT0056] Omar S. 1997. The role of rock-phosphate-solubilizing fungi and vesicular–arbusular-mycorrhiza (VAM) in growth of wheat plants fertilized with rock phosphate. World Journal of Microbiology and Biotechnology 14, 211–218.

[CIT0057] Pantigoso HA , Newberger D, Vivanco JM. 2022. The rhizosphere microbiome: plant–microbial interactions for resource acquisition. Journal of Applied Microbiology 133, 2864–2876. 36648151 10.1111/jam.15686 PMC9796772

[CIT0058] Park JM , Kim B, Cho Y-C, Lee B-H, Hong JW, You Y-H. 2021. Rhizoplane and rhizosphere fungal communities of geographically isolated Korean Bellflower (*Campanula takesimana* Nakai). Biology 10, 138. 33578742 10.3390/biology10020138 PMC7916508

[CIT0059] Parvin S , Van Geel M, Yeasmin T, Verbruggen E, Honnay O. 2020. Effects of single and multiple species inocula of arbuscular mycorrhizal fungi on the salinity tolerance of a Bangladeshi rice (*Oryza sativa* L.) cultivar. Mycorrhiza 30, 431–444. 32367433 10.1007/s00572-020-00957-9

[CIT0060] Prajapati K , Modi H. 2012. Isolation and characterization of potassium solubilizing bacteria from ceramic industry soil. CIBTech Journal of Microbiology 1, 8–14.

[CIT0061] Price MN , Dehal PS, Arkin AP. 2010. FastTree 2—approximately maximum-likelihood trees for large alignments. PLoS One 5, e9490. 20224823 10.1371/journal.pone.0009490 PMC2835736

[CIT0062] Radhakrishnan R , Kang S, Baek I, Lee I. 2014. Characterization of plant growth-promoting traits of Penicillium species against the effects of high soil salinity and root disease. Journal of Plant Interactions 9, 754–762.

[CIT0063] R Core Team. 2022. R: a language and environment for statistical computing. Vienna, Austria: R Foundation for Statistical Computing.

[CIT0064] Reid TE , Kavamura VN, Abadie M, Torres-Ballesteros A, Pawlett M, Clark IM, Harris J, Mauchline TH. 2021. Inorganic chemical fertilizer application to wheat reduces the abundance of putative plant growth-promoting rhizobacteria. Frontiers in Microbiology 12, 642587. 33776974 10.3389/fmicb.2021.642587 PMC7991844

[CIT0065] Rognes T , Flouri T, Nichols B, Quince C, Mahé F. 2016. VSEARCH: a versatile open source tool for metagenomics. PeerJ 4, e2584. 27781170 10.7717/peerj.2584 PMC5075697

[CIT0066] Saitou N , Nei M. 1987. The Neighbor–Joining method: a new method for reconstructing phylogenetic trees. Molecular Biology and Evolution 4, 406–425. 3447015 10.1093/oxfordjournals.molbev.a040454

[CIT0067] Santos-Torres M , Romero-Perdomo F, Mendoza-Labrador J, Gutiérrez AY, Vargas C, Castro-Rincon E, Caro-Quintero A, Uribe-Velez D, Estrada-Bonilla GA. 2021. Genomic and phenotypic analysis of rock phosphate-solubilizing rhizobacteria. Rhizosphere 17, 100290.

[CIT0068] Saravanan VS , Subramoniam SR, Raj SA. 2003. Assessing *in vitro* solubilization potential of different zinc solubilizing bacterial (ZSB) isolates. Brazilian Journal of Microbiology 35, 121–125.

[CIT0069] Schmitz L , Yan Z, Schneijderberg M, et al 2022. Synthetic bacterial community derived from a desert rhizosphere confers salt stress resilience to tomato in the presence of a soil microbiome. The ISME Journal 16, 1907–1920. 35444261 10.1038/s41396-022-01238-3 PMC9296610

[CIT0070] Selbmann L , Stoppiello GA, Onofri S, Stajich JE, Coleine C. 2021. Culture-dependent and amplicon sequencing approaches reveal diversity and distribution of black fungi in Antarctic cryptoendolithic communities. Journal of Fungi 7, 213. 33809619 10.3390/jof7030213 PMC8001563

[CIT0071] Sembiring M , Sakiah J, Wahyuni M. 2018. The inoculation of mycorrhiza and *Talaromyces pinophilus* toward the improvement in growth and phosphorus uptake of oil palm seedlings (*Elaeis guineensis* Jacq) on saline soil media. Bulgarian Journal of Agricultural Science 24, 617–622.

[CIT0072] Sharma S , Rathod ZR, Jain R, Goswami D, Saraf M. 2023. Strategies to evaluate microbial consortia for mitigating abiotic stress in plants. In: Maheshwari DK, Dheeman S, eds. Microorganisms for sustainability. Singapore: Springer Nature, 171–203.

[CIT0073] Shcherbak I , Millar N, Philip Robertson GP. 2014. Global metaanalysis of the nonlinear response of soil nitrous oxide (N_2_O) emissions to fertilizer nitrogen. Biological Sciences 111, 9199–9204. 10.1073/pnas.1322434111 PMC4078848 24927583

[CIT0074] Silva BM , Prados-Rosales R, Espadas-Moreno J, Wolf JM, Luque-Garcia JL, Gonçalves T, Casadevall A. 2014. Characterization of *Alternaria infectoria* extracellular vesicles. Medical Mycology 52, 202–210. 24576997 10.1093/mmy/myt003 PMC4294692

[CIT0075] Silva JV , Jaleta M, Tesfaye K, et al 2023. Pathways to wheat self-sufficiency in Africa. Global Food Security 37, 100684. 37351552 10.1016/j.gfs.2023.100684 PMC10282895

[CIT0076] Simonin M , Dasilva C, Terzi V, Ngonkeu ELM, Diouf D, Kane A, Béna G, Moulin L. 2020. Influence of plant genotype and soil on the wheat rhizosphere microbiome: evidences for a core microbiome across eight African and European soils. FEMS Microbiology Ecology 96, fiaa067. 32275297 10.1093/femsec/fiaa067

[CIT0077] Singh H , Reddy MS. 2011. Effect of inoculation with phosphate solubilizing fungus on growth and nutrient uptake of wheat and maize plants fertilized with rock phosphate in alkaline soils. European Journal of Soil Biology 47, 30–34.

[CIT0078] Tabatabai MA , Bremner JM. 1969. Use of p-nitrophenyl phosphate for assay of soil phosphatase activity. Soil Biology and Biochemistry 1, 301–307.

[CIT0079] Tamburini G , Bommarco R, Wanger TC, Kremen C, van der Heijden MGA, Liebman M, Hallin S. 2020. Agricultural diversification promotes multiple ecosystem services without compromising yield. Science Advances 6, 1715. 10.1126/sciadv.aba1715 PMC7673676 33148637

[CIT0080] Tamura K , Stecher G, Kumar S. 2021. MEGA11: molecular evolutionary genetics analysis Version 11. Molecular Biology and Evolution 38, 3022–3027. 33892491 10.1093/molbev/msab120 PMC8233496

[CIT0081] Tarroum M , Romdhane WB, Al-Qurainy F, Mohamed AAA, Al-Doss A, Fki L, Hassairi A. 2022. A novel PGPF *Penicillium olsonii* isolated from the rhizosphere of *Aeluropus littoralis* promotes plant growth, enhances salt stress tolerance, and reduces chemical fertilizers inputs in hydroponic system. Frontiers in Microbiology 13, 996054. 36386667 10.3389/fmicb.2022.996054 PMC9648140

[CIT0082] Tian X , Engel BA, Qian H, Hua E, Sun S, Wang Y. 2021. Will reaching the maximum achievable yield potential meet future global food demand? Journal of Cleaner Production 294, 126285–126526.

[CIT0083] Umesha S , Singh PK, Singh RP. 2018. Microbial biotechnology and sustainable agriculture. In: Singh RL, Monda S, eds. Biotechnology for sustainable agriculture. Sawston: Woodhead Publishing, 185–205.

[CIT0084] Vassilev N , Vassileva M, Lopez A, Martos V, Reyes A, Maksimovic I, Eichler-Löbermann B, Malusà E. 2015. Unexploited potential of some biotechnological techniques for biofertilizer production and formulation. Applied Microbiology and Biotechnology 99, 4983–4996. 25957155 10.1007/s00253-015-6656-4

[CIT0085] Wakelin SA , Gupta VV, Harvey PR, Ryder MH. 2007. The effect of *Penicillium* fungi on plant growth and phosphorus mobilization in neutral to alkaline soils from southern Australia. Canadian Journal of Microbiology 53, 106–115. 17496956 10.1139/w06-109

[CIT0086] Wickham H. 2016. ggplot2: elegant graphics for data analysis. New York: Springer-Verlag.

[CIT0087] Wieland G , Neumann R, Backhaus H. 2001. Variation of microbial communities in soil, rhizosphere, and rhizoplane in response to crop species, soil type, and crop development. Applied and Environmental Microbiology 67, 5849–5854. 11722945 10.1128/AEM.67.12.5849-5854.2001 PMC93382

[CIT0088] Xiao HQ , Chi RA, Huang XH, Zhang WX, Qiu GZ, Wang DZ. 2008. Optimization for rock phosphate solubilization by phosphate-solubilizing fungi isolated from phosphate mines. Ecological Engineering 33, 187–193.

[CIT0089] Yao Y , Li G, Lu Y, Liu S. 2023. Modelling the impact of climate change and tillage practices on soil CO_2_ emissions from dry farmland in the Loess Plateau of China. Ecological Modelling 478, 110276.

[CIT0090] York LM , Carminati A, Mooney SJ, Ritz K, Bennett MJ. 2016. The holistic rhizosphere: integrating zones, processes, and semantics in the soil influenced by roots. Journal of Experimental Botany 67, 3629–3643. 26980751 10.1093/jxb/erw108

[CIT0091] Zambrano-Mendoza JL , Sangoquiza-Caiza CA, Campaña-Cruz DF, Yánez-Guzmán CF. 2021. Use of biofertilizers in agricultural production.In: Ahmad F, Sultan M, eds. Technology in agriculture. Intech Open. doi: https://doi.org/10.5772/intechopen.98264.

[CIT0092] Zhang X , Rajendran A, Grimm S, Sun X, Lin H, He R, Hu B. 2023. Screening of calcium- and iron-targeted phosphorus solubilizing fungi for agriculture production. Rhizosphere 26, 100689.

[CIT0093] Zhao J , Yang Y, Zhang K, Jeong J, Zeng Z, Zang H. 2020. Does crop rotation yield more in China? A meta-analysis. Field Crops Research 245, 107659.

[CIT0094] Zheng H , Qiao M, Xu J, Yu Z. 2021. Culture-based and culture-independent assessments of endophytic fungal diversity in aquatic plants in Southwest China. Frontiers in Fungal Biology 2, 692549. 37744110 10.3389/ffunb.2021.692549 PMC10512276

