# Supplementary material for: Unlocking the agro-physiological potential of wheat rhizoplane fungi under low P conditions using a niche-conserved consortium approach
Source: J Exp Bot. 2025 Feb 26;76(8):2320–37. doi: 10.1093/jxb/eraf042 (PMC12116180; doi:10.1093/jxb/eraf042)
Supplement: eraf042_suppl_Supplementary_Figure_S1_Tables_S1-S4 [file eraf042_suppl_supplementary_figure_s1_tables_s1-s4.pdf]

Supplementary information

Unlocking the agro-physiological potential of wheat rhizoplane fungi using a niche-conserved consortium construction approach with low P conditions

Journal of Experimental Botany

Brahim Benbrik*[1], Tessa E. Reid[2], Dounia Nkir[1], Hicham Chaouki[1], Yassine Aallam[1], Ian M. Clark[2], Tim H. Mauchline[2], Jim Harris[3], Mark Pawlett[3], Abdellatif Barakat[1,4], Zineb Rchiad[5], Adnane Bargaz*[1]

[1] AgroBiosciences Program, College of Agriculture and Environmental Sciences, Mohammed 6 Polytechnic University, Ben Guerir, Morocco.

[2] Sustainable Soils and Crops, Rothamsted Research, Harpenden, United Kingdom.

[3] Environment and Agrifood, Faculty of Engineering and Applied Sciences, Cranfield University, Cranfield MK43 0AL, United Kingdom.

[4] IATE, Univ Montpellier, INRAE, Agro Institut. 2, Place Pierre Viala, 34060 Montpellier, France.

[5] Biosciences Division, CoreLabs, Mohammed 6 Polytechnic University, Ben Guerir, Morocco.

Corresponding author (s):

Adnane Bargaz: Adnane.bargaz@um6p.ma

ORCID

Braim Benbrik : https://orcid.org/0000-0001-7739-0711

Adnane Bargaz: https://orcid.org/0000-0003-2850-027X

Physical mailing address:

Mohammed 6 Polytechnic University Lot 660, Hay Moulay Rachid Ben Guerir, 43150, Morocco; phone. Fixe: +212 525 073 100. Fax: +212 525 073 134. E-mail: contact@um6p.ma

This file includes:

Supplementary Fig. S1

Supplementary Tables S1-S4

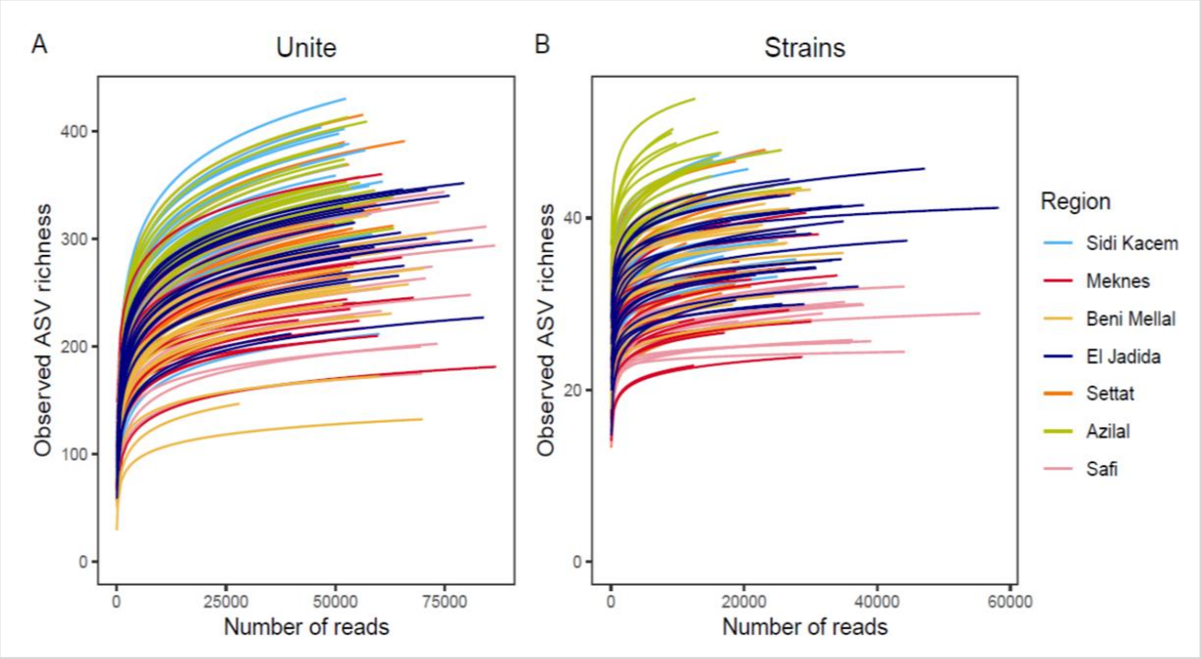

Supplementary Fig. S1. Rarefaction curves showing the number of fungal ASVs observed against the number of sequences sampled for (A) all ASVS (4,568) classified by Unite and (B) ASVs that were identified as isolated fungal strains (231 ASVs) from isolate ITS sequences.

Supplementary Table S1. Fungal composition of zone (niche-conserved), intra-region FC, and the inter-regions (global) FC. FC-oriented approach allowed to construct 28 zone FC (FC_1 – FC_{28}) as many as the number of sampling zones. Additionally, 7 intra-region FC were constructed (FC_{R1} – FC_{R7}) as well as one global FC (FC_G). Prior to FC construction, antagonistic test was done between each zone fungal isolates, intra-region fungal isolates and all fungal isolates respectively, to remove non compatible isolates within each FC. According to ITS fungal identification, the literature, and the 20-day old wheat seedling test, the barred isolates (F_8, F_{10} and F_{19}) were considered as pathogenic, and were eliminated from the FC. The resulting FC with a single individual isolate were then discarded, generating a total of 23 FC including fifteen niche-conserved (or zone FC), seven intra-region (FC R1 – FCR7), and one inter-regions "global" (FCG) FC.

**Region**	**Sidi Kacem (SK)**				**Meknes (MK)**				**El Jadida (EJ)**				**Settat (ST)**				**Beni Mellal (ST)**				**Safi (SA)**				**Azilal (AZ)**			
**Zone (niche)**	Sidi Kacem	Sidi Slimane	Dar Laaouinat	Labhalil	Ait Lhakkour	Meknes	Ait Ouhssine	El Borj	Rdaidat	Oulad Bouanane	Oulad Saad	Sidi Smail	Kh. Chaouia	Zakkach	Oulad Amer	Oulad Fdel	Ouaramane	Afourar	Sidi Jaber	Fkih Ben Saleh	El Haddada	Oulad Taleb	Oulad Hammou	Bouguedra	Lounasda	Zalgane	Maskaoun	Eddachra
**Zone Consortia ID**	FC_1	FC_2	FC_3	FC_4	FC_5	FC_6	FC_7	FC_8	FC_9	FC_{10}	FC_{11}	FC_{12}	FC_{13}	FC_{14}	FC_{15}	FC_{16}	FC_{17}	FC_{18}	FC_{19}	FC_{20}	FC_{21}	FC_{22}	FC_{23}	FC_{24}	FC_{25}	FC_{26}	FC_{27}	FC_{28}
**Number of fungal isolates per zone**	1	2	2	3	1	2	1	2	2	1	1	0	2	2	2	1	2	2	1	1	2	2	1	1	1	3	1	3
**Fungal composition per zone (niche-conserved FC)**	F1<br>~~F8*~~	F2<br>F9	F2<br>F3	F4<br>F7<br>F17	F3	F5<br>F13	F14<br>~~F19*~~	F6<br>F15	F1<br>F3<br>~~F10*~~	F7<br>~~F8*~~	F13<br>F8	–	F9<br>~~F10*~~<br>F2	F11<br>F12	F1<br>F6	F15	F11<br>F9	F12<br>F13	F14	F18<br>~~F19*~~	F15<br>F16	F7<br>F3	F17	F18	~~F19*~~<br>F20	~~F10*~~<br>F16<br>F17	F20	F2<br>F4<br>F7
**Fungal composition of intra-region FC (FC_R) ****	F1, F2, F3, F9<br>(FC_{R1})				F3, F5, F6, F14, F15<br>(FC_{R2})				F1, F3<br>(FC_{R3})				F1, F6, F9, F11, F12, F15<br>(FC_{R4})				F9, F11, F12, F14, F18<br>(FC_{R5})				F3, F15, F16, F17, F18<br>(FC_{R6})				F2, F16, F20<br>(FC_{R7})			
**Fungal composition of global inter-regions FC (FC_G) ****	F1, F2, F3, F5, F6, F9, F11, F12, F14, F15, F16, F18, F20<br>(FC_G)																											

** Pathogenic isolates such F8 were discarded from constructed FC.*
*** Antagonism test was performed within each FC and antagonistic (non-compatible) isolates were then discarded from constructed FC.*

Supplementary Table S2. ASVs classified to genus level for: 1) all ASVs classified by Unite, 2) the same ASVs identified as isolates but classified by Unite, and 3) ASVs that were identified as isolates. In total, 311 genera were classified by the Unite database. Only the genera that are also genera from the isolates are shown. '<NA> ': when ASVs are classified but not to genus level; 'unidentified': when the genus for the ASVs in unidentified. For full classification of the same ASVs classified by different databases, see Supplementary Data S1.

**All ASVs**		**Same ASVs classified by different databases**			
Genera classified by the Unite database	ASV no.	Genera classified by the Unite database	ASV no.	Genera identified by the isolate database	ASV no.
Alternaria	117	*Alternaria*	88	*Alternaria*	88
Aspergillus	85	*Aspergillus*	42	*Aspergillus*	41
Penicillium	76			*Penicillium*	1
Fusarium	45	*Fusarium*	27	*Fusarium*	42
Talaromyces	27	*Talaromyces*	1	*Talaromyces*	1
Actinomucor	10	*Actinomucor*	10	*Actinomucor*	10
Rhizopus	4	*Rhizopus*	4	*Rhizopus*	4
Didymella	2	*Didymella*	2	*Neodidymelliopsis*	44
<NA>	1099	<NA>	48		
unidentified	920	unidentified	9		
Other genera (303)	2008				
**Total**	**4393**	**Total**	**231**	**Total**	**231**

Supplementary Table S3. Above-ground plant parameters (SH, Leaf area and CCI), rhizosphere available P, shoot K and N uptake of 30-day and 70-day old wheat plants inoculated with FC versus uninoculated treatments (Ortho-P, RP and P_0) under RP supply. CCI: Chlorophyl content index, SH: shoot height, Ortho-P: fertilized with orthophosphates, RP: fertilized with phosphate rock, and P_0: unfertilized and uninoculated, FC: zone FC, FC_{R1}-FC_{R7}: intra-region FC, FC_G: global (inter-regions) FC.

	30-day old seedling		70-day old seedling					
Treatments	SH (cm)	CCI	SH (cm)	Leaf area	CCI	Rhizosphere available-P (mg/kg)	Shoot K uptake (mg K / plant)	Shoot N uptake (mg N/ plant)
Ortho-P	17.5 def	1.7 abc	32.1 a-e	30.9 ab	6.1 d-i	53.3 a	14.0 bcd	14.7 c-g
RP	13.7 fgh	1.3 b-e	30.4 b-f	21.4 a-e	6.0 d-i	27.6 hi	10.6 f-j	15.3 c-f
P_0	10.5 hi	1.1 de	19.2 jk	16.3 c-f	1.2 ij	13.4 k	8.7 jkl	7.8 h
FC2	17.0 def	1.2 cde	26.5 d-i	27.0 abc	1.9 g-j	36.3 fg	13.5 b-e	15.1 c-f
FC3	15.0 efg	1.2 e	28.7 b-g	25.0 a-e	2.3 f-j	40.4 c-f	13.1 c-g	16.1 cde
FC4	15.0 efg	1.3 b-e	23.5 f-k	26.4 a-d	1.6 hij	38.9 def	9.4 ijk	10.1 e-h
FC6	18.2 cde	1.6 abc	32.0 a-e	29.0 abc	9.8 bcd	52.4 a	11.6 d-i	17.2 bcd
FC8	19.2 bcd	1.6 abc	33.0 a-d	31.7 ab	12.3 abc	54.3 a	19.3 a	23.0 ab
FC9	21.0 abc	1.6 abc	34.8 abc	34.5 a	14.0 ab	53.8 a	22.0 a	19.7 abc
FC13	14.7 efg	1.2 cde	25.8 d-j	29.4 c-f	6.5 d-h	46.4 b	13.6 b-e	16.3 cd
FC14	23.3 a	2.0 a	38.6 a	35.4 a	14.9 a	53.0 a	19.7 a	23.2 ab
FC15	13.7 fgh	1,6 a-d	29.4 b-f	9,5 f	5.4 d-j	44.6 bc	11.5 d-i	13.2 d-h
FC17	22.3 ab	1.8 ab	35.5 ab	32.2 ab	14.4 a	55.4 a	19.6 a	23.6 a
FC18	17.2 def	1.5 b-d	21.2 h-k	24.3 a-e	1.3 ij	44.9 bc	7.1 kl	8.4 gh
FC21	15.0 efg	1.2 cde	22.2 g-k	27.3 abc	1.0 j	42.6 bcd	11.6 d-i	12.3 d-h
FC22	9.3 i	1,2 cde	27.8 c-h	21.8 a-e	3.1 e-j	32.5 gh	12.9 c-h	13.5 c-g
FC26	13.2 gh	1.3 b-e	29.1 b-g	19.9 b-f	3.4 e-j	39.8 c-f	14.9 bc	15.2 c-f
FC28	14.7 efg	1.3 b-e	27.5 d-i	16.3 c-f	1.8 g-j	38.5 def	11.1 e-j	12.5 d-h
FCR1	13.2 gh	1,1 de	27.7 c-i	19.9 b-f	4.1 e-j	37.1 d-g	10.3 hij	12.4 d-h
FCR2	13.7 fgh	1.2 cde	27.2 d-i	25.0 a-e	2.6 f-j	23.4 ij	10.4 g-j	9.4 f-h
FCR3	15.0 efg	1.2 cde	18.7 k	25.9 abc	1.4 ij	21.8 i	6.9 kl	7.6 h
FCR4	16.5 d-g	1.5 b-d	20.7 ijk	27.3 abc	2.0 f-j	31.9 gh	6.4 l	8.6 gh
FCR5	16.3 d-g	1.3 b-e	25.5 e-k	13.2 a-f	6.6 d-g	42.2 b-e	11.2 e-j	16.1 cde
FCR6	13.7 fgh	1.5 b-d	30.0 b-f	12.2 ef	9.4 cd	46.2 b	15.8 b	17.6 a-d
FCR7	15.0 efg	1.5 b-d	29.4 b-f	19.9 def	6.8 def	36.8 efg	13.0 c-g	18.4 a-d
FCG	15.0 efg	1.2 cde	31.1 b-r	25.7 a-d	7.5 de	37.3 d-g	13.2 b-f	17.4 bcd

Values are means ± SD (n = 3). Different letters indicate values that are statistically different at $p < 0.05$.

Supplementary Table S4. Pearson correlation between soluble P, pH and gluconic acid concentration of fungal isoltaes (F1 to F20) grown in NBRIP liquid medium supplemented with TCP.

	Soluble P	pH	Gluconic acid
Soluble P	-	r^2= - 0.865** ($p < 0.001$)	r^2 = 0.818** ($p < 0.001$)
pH	r^2= - 0.865** ($p < 0.001$)	-	r^2 = - 0.854** ($p < 0.001$)
Gluconic acid	r^2 = 0.818** ($p < 0.001$)	r^2 = - 0.854** ($p < 0.001$)	-

** *Correlation is significant at the 0.01 level (2-tailed).*
